# 2,4-Thiazolidinedione Treatment Improves the Innate Immune Response in Dairy Goats with Induced Subclinical Mastitis

**DOI:** 10.1155/2017/7097450

**Published:** 2017-06-27

**Authors:** Fernanda Rosa, Johan S. Osorio, Erminio Trevisi, Francisco Yanqui-Rivera, Charles T. Estill, Massimo Bionaz

**Affiliations:** ^1^Department of Animal and Rangeland Sciences, Oregon State University, Corvallis, OR 97331, USA; ^2^Istituto di Zootecnica, Facoltà di Scienze Agrarie, Alimentari e Ambientali, Università Cattolica del Sacro Cuore, 29122 Piacenza, Italy

## Abstract

Mastitis is a major disease in dairy cows resulting in significant economic losses. In vitro works suggest that ruminants peroxisome proliferator-activated receptor gamma (PPAR*γ*) can aid in improving the response to mastitis and can control milk fat synthesis. The objectives of the present experiment were to test if treatment with the putative PPAR*γ* agonist 2,4-thiazolidinedione (TZD) improves (1) the response to subclinical mastitis and (2) milk fat production. Lactating goats received daily injections of 8 mg/kg BW of TZD or saline for 3 weeks. After one week of TZD injection, half of the goats in each group received intramammary infusion of* Strep. uberis* or saline in both halves for a total of 4 groups (*n* = 6/group). TZD treatment did not affect milk fat but had positive effect on milk somatic cells count, blood nonesterified fatty acids, inflammatory markers, and liver function. TZD significantly increased myeloperoxidase but did not affect leukocytes phagocytosis or insulin. TZD increased adipocytes size and had minor effect on expression of PPAR*γ* target genes in mammary epithelial cells but not in adipose tissue. Overall, TZD ameliorated the response to intramammary infection but the effect on milk fat synthesis and expression of related transcripts was less than expected.

## 1. Introduction

Mastitis is an inflammatory response of the udder to infection (usually bacteria) with detrimental consequences to animal well-being and milk quantity and quality [1–3]. It has been estimated that the cost of mastitis in the US dairy industry is ca. $2 billion annually or 11% of total U.S. milk sales, with an average of ca. $179/cow [4, 5].

Mastitis may be clinical or subclinical. Subclinical mastitis can be detected only by the measurement of somatic cells count (SCC) in milk, which increases several fold compared to milk from healthy mammary glands. The high SCC during subclinical mastitis negatively affects milk quality and reduces overall milk yield. It has been estimated that subclinical mastitis costs $130/cow per year [1]. Considering approximately 9 million dairy cows in the US, the cost can be over $1 billion/year. Therefore, prevention of subclinical mastitis is a priority for the dairy industry.

From a nutritional standpoint it is becoming evident that adequate levels of dietary antioxidants, particularly vitamin E, selenium, zinc, and vitamin A, reduce the incidence of environmental mastitis in dairy cows [3, 6, 7]. Besides providing protection from oxidative stress, several of these compounds enhance the mammary response to mastitis [6]. It is becoming increasingly evident that dietary compounds can profoundly alter the host's response via effect on the transcriptome [8–10]. Nutrigenomics is the discipline that studies such effect [11] and it is revolutionizing the field of nutrition [12]. Dietary compounds can affect the transcriptome via binding to transcription factors (TF) [11]. Peroxisome Proliferator-Activated Receptors (PPARs) are, among TF, the most interesting from a nutrigenomic standpoint [11, 13].

PPARs are nuclear receptors and members of the nuclear hormone receptor superfamily that work as TF. PPARs form a heterodimer with retinoid-X receptor (RXR) and, when activated by natural or synthetic agonists, modulate transcription by binding to a specific DNA sequence termed PPAR response element (PPRE) inducing the transcription of target genes [14]. Three PPAR isotypes denominated PPAR*α*, PPAR*β*/*δ*, and PPAR*γ* have been identified in several species, including bovine [13]. Among the three PPAR isotypes the PPAR*γ* is highly expressed in bovine white adipose tissue but its expression is also relatively abundant in mammary tissue [13]. Long-chain fatty acids (LCFA) are among the most potent agonists of bovine PPAR suggesting the possibility of dietary interventions to improve the response to diseases in dairy cows via activation of PPAR [13].

Furthermore, PPAR*γ* plays a crucial role in the regulation of lipid and glucose metabolism [14] by regulating adipogenesis and lipogenesis [15] and by insulin-sensitizing effects [16]. The same functions of PPAR*γ* appear to be conserved in ruminants [13].

In mammary tissue of dairy cows expression of PPAR*γ* is upregulated from late pregnancy to early lactation [17] indicating a role of this nuclear receptor in controlling milk fat synthesis. This was supported by in vitro studies performed in cattle and goats [18–20] but not in mice [21], suggesting that the role of PPAR*γ* in milk fat synthesis is likely ruminant-specific [13].

Besides a role in the regulation of milk fat synthesis, PPAR*γ* may have also a positive role in the host's response to mammary infection, as reviewed recently [11, 22]. In nonruminants, PPAR*γ* is known to have anti-inflammatory effects [23]. In bovine primary mammary epithelial cells (bMEC) the activation of PPAR*γ* by several agonists caused downregulation of several proinflammatory cytokines and increased expression of the Chemokine (C-C Motif) Ligand 2 and Tumor Necrosis Factor alpha [24]. In contrast, activation of PPAR*γ* by a natural agonist markedly enhanced the expression of both interleukin 8 and Chemokine (C-X-C motif) Ligand 6 but had no effect on other cytokines [24]. Interleukin 8 is the strongest chemoattractant for neutrophils and higher production is desirable for a quick response to intramammary infection [25, 26]. In addition, intramammary infusion with* Strep. uberis* had a concomitant reduction of milk fat synthesis and downregulation of PPAR pathway in dairy cows [27]. The PPAR*γ* may also play a role in tissue regeneration after mastitis because in monogastrics all PPAR isotypes play an important role in wound reepithelialization [28].

Our long-term goal is to improve the overall health and performance of dairy animals via nutrigenomic approaches. We have hypothesized that activation of various PPAR isotypes by LCFA can improve the transition from pregnancy to lactation in dairy cows [13]. The present work focuses on PPAR*γ* and its role on mammary response to infection and milk fat synthesis. Our hypotheses are that activation of PPAR*γ* (1) improves the response to mammary infection and (2) augments synthesis of milk fat. To assess these hypotheses we performed a proof-of-concept study using a putative synthetic PPAR*γ* agonist with the objective to test if activation of PPAR*γ* prior to and during induced subclinical mastitis (1) improves the response to mastitis and (2) affects milk fat synthesis in dairy goats.

## 2. Materials and Methods

### 2.1. Experiment Design and Animal Management

**Figure 1:**
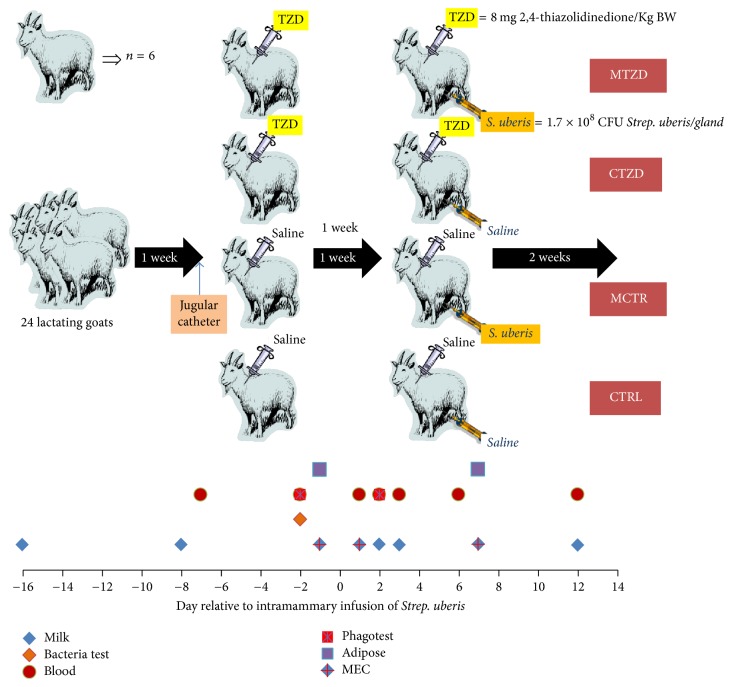
Overview of the experimental design with time points for collection of samples. MEC = mammary epithelial cell isolation.

The Institutional Animal Care and Use Committee (IACUC) of Oregon State University approved all procedures for this study (protocol # 4448). An overview of the experimental design is depicted in Figure 1. Twenty-four lactating Saanen goats (mean ± SD; age 5.1 ± 0.6 years old, 3.6 ± 0.6 lactations, 156 ± 14 day in milk, 68.1 ± 7.6 kg of BW, and 1.6 ± 0.5 body condition score [1–5 scale]), negative to a milk bacterial analysis, were purchased from a commercial farm (Tumalo Farm, Bend, Oregon, USA) and housed in the Oregon State University Sheep Center facility. One week of adaptation to the new environment was allowed prior to starting the experiment. The goats were assigned to treatments in a randomized block design (blocked by body condition score, body weight, milk yield, and milk components) and housed in 4 separate pens (6 goats/pen). Animals were fed twice a day (8 AM and 6 PM) with a diet similar to what was fed at the farm of origin with ad libitum hay (approx. 50% orchard hay and 50% alfalfa). The goats received approximately 150 g of a commercial grain goat mix (Kountry Buffet, PayBack, Harrisburg, OR) during milking. The goats were milked once a day at 8 AM in a stanchion using a portable milking machine. Teats of the goats were predipped before milking and postdipped after milking using 0.5% iodine solution.

### 2.2. Treatments

After the week of adaptation, goats were fitted with an indwelling jugular catheter (Cat# 017376, Henry Schein, USA) with an extension set (Cat# 005642, Henry Schein). Originally the catheter was kept in place and hidden from the goats using Elastikon stretch bandage (Cat# 000925, Henry Schein); after one week a bandage of VetWrap 4′′ was used for practicality. The catheter was flushed twice a day using 10 mL of heparinized saline (2 i.u./mL). The day after catheter insertion, 12 goats received daily injection of 8 mg/kg of BW of 2,4-thiazolidinedione (TZD; Cat# 375004, Sigma-Aldrich, USA) in 10 mL sterile physiological saline (VINV-SALN-1000, Henry Schein) throughout the entire study period (20 days). The other goats (*n* = 12; CTR) received 10 mL of physiological saline. The daily injection was performed at 12 PM. The dose of TZD was selected based on the reported efficacy of 4 mg/Kg BW in dairy cows [29] and the faster drug clearance of dairy goats [30] that requires typically doubling the dose of many drugs for this species compared to bovine.

### 2.3. Intramammary Infusion of* Streptococcus uberis*

After one week of TZD or saline injection, half of the goats in each treatment received an intramammary infusion (IMI) of 1.7 × 10^8^* Streptococcus uberis (Strep. uberis)* in 10 mL sterile physiological saline in each of the mammary halves (the groups were mastitis control or MCTR and mastitis TZD or MTZD) following a previously published protocol [31]. The remaining goats received an intramammary infusion of 10 mL sterile physiological saline (control saline or CTRL and control TZD or CTZD) in each half gland. The aliquots of* Strep. uberis* in 1.5 mL sterile vials were provided by the laboratory of Peggy Dearing, College of Veterinary Medicine, Oregon State University. The intramammary infusion was performed just after the morning milking after carefully cleaning the teat ends with individual moistened towels and disinfecting them with swabs containing 70% ethanol. The infusion was performed with the aid of a disposable sterile urinary catheter (TomCat, USA). After IMI, each mammary half was thoroughly massaged upward into the gland cistern for approximately 20 s to distribute the inoculum.

The* Strep. uberis* used in the present experiment was isolated from a mastitic cow and the strain determined using DNA isolated from the bacteria using a DNA Clean & Concentrator-5 (cat# D4013, Zymo Research, Tustin, CA) and submitted for Sanger sequencing at Center for Genome Research and Biocomputing at Oregon State University. The sequencing result was blasted against all bacteria sequences using BLASTN 2.4.0 at National Center for Biotechnology Information. The sequence had 89% identity with* Strep. uberis* strain 0140J (File S1 in Supplementary Material available online at https://doi.org/10.1155/2017/7097450).

### 2.4. Follow-Up Study

Two animals in the MTZD group ceased to produce milk after IMI and had increase in temperature >41°C. For this reason the animals had to be removed from the study; thus, the MTZD groups had only 4 animals. This event raised the possibility that the cause of agalactia was the interaction between TZD injection and IMI. In order to test this we performed a follow-up study using 4 animals from the CTZD and 4 animals from the CTRL. The study followed the same protocols as the main study. In the present paper the data from the follow-up study are not reported, with the exception of frequency of agalactia.

### 2.5. Measurements and Sample Collection

Milk was collected aseptically from both halves of the mammary for bacterial analysis at the time of purchase, 3 days prior to IMI, and just before IMI. Before sampling, the teat was treated with teat dip solution and cleaned using disposable paper towels. The orifice of the teat was disinfected with swabs containing 70% ethanol, first stripped milk was discarded, and approximately 1 mL of milk was collected in sterile 1.5 mL tubes. The samples were immediately put on ice and shipped within 4 hours to the Ag Health Laboratories, Inc. (Sunnyside, WA), for bacterial culture blood agar plates. All samples were negative to* Strep. uberis*. To ascertain SCC status a California Mastitis Test was performed at the last milking prior to IMI.

Milk yield was recorded daily throughout the experiment. Milk samples were collected for component analysis 5 days prior to treatment assignment and then the day before starting TZD injections, the day before IMI, and 1, 2, 3, 5, 7, and 12 d after IMI. Samples were collected and shipped to the Willamette National Dairy Herd Information Association (DHIA, Salem, OR) using 10 mL vials provided by the DHIA containing Bronopol. SCC, lactose, fat, and protein were measured in milk. The energy corrected milk (ECM) was calculated using the equation (0.327 × kg of milk) + (12.95 × kg of fat) + (7.65 × kg of protein) [32].

Rectal temperature was checked daily using a rectal thermometer prior to and after IMI and every hour during the first 6 h after IMI and approximately every 8 h until 114 h after IMI. Body weight (BW) was recorded weekly throughout the study.

Blood samples were collected prior to the morning feeding from the jugular vein using a 20-gauge BD Vacutainer needle (Becton Dickinson, Franklin Lakes, NJ). The blood was collected just before starting the TZD injections (i.e., 7 days prior to IMI or baseline), 2 days prior to IMI (5 days into TZD injections), every day during the first 3 days after IMI (i.e., 24 h, 48 h, and 72 h after IMI), and at 6 and 12 d after IMI. Samples were collected into evacuated tubes (10 mL, BD Vacutainer®, Becton Dickinson, Franklin Lakes, NJ) containing either serum clot activator or sodium heparin. After blood collection, tubes containing sodium heparin were placed on ice, while the tubes with clot activator were kept at room temperature (~30 min) until centrifugation in a C3 Select centrifuge (LW Scientific) and frozen at −20°C until being analyzed.

### 2.6. Blood Metabolites and Inflammatory Markers

Aliquots of plasma and serum were shipped on dry ice to the Istituto di Zootecnica, Università Cattolica del Sacro Cuore, Piacenza, Italy, for metabolic and inflammatory profiling. Blood samples were analyzed for 19 parameters. These include the metabolic parameters glucose, cholesterol, urea, calcium, magnesium, nonesterified fatty acids (NEFA), triacylglycerol (TG), *β*-hydroxybutyric acid (BHBA), and retinol and the inflammatory-related parameters albumin, haptoglobin, ceruloplasmin, paraoxonase, myeloperoxidase, total bilirubin, total reactive oxygen metabolites (ROMt), zinc, and *α*-tocopherol plus the liver parameter gamma-glutamyl transferase (*γ*GT). The analyses were performed using a clinical autoanalyzer (ILAB 650, Instrumentation Laboratory, Lexington, MA) and HPLC following procedures described previously [33–35].

### 2.7. Insulin, QUICKI, and RQUICKI

Concentration of insulin was analyzed in plasma 5 days after TZD injection (i.e., −2 d relative to IMI) and 3 d after IMI using a commercial ELISA assay kit (NeoScientific Cat# GTI0011) following the manufacturer instruction. The Quantitative Insulin Sensitivity Check Index (QUICKI; [36]) and the Revised QUICKI (RQUICKI; [37]) were calculated as follows:

(1) QUICKI=1log⁡fasting  insulin  μU/mL+log⁡fasting  glucose  mg/dLRQUICKI=1log⁡fasting  insulin  μU/mL+log⁡fasting  glucose  mg/dL+log⁡NEFA  mmol/l.

### 2.8. Phagocytosis and % Granulocytes

The phagocytic capacity of leukocytes isolated from 100 *μ*L heparinized whole blood was determined at −2 and 1 d relative to IMI using Phagotest kit (Glycotope Biotechnology, Heidelberg, Germany) following the manufacturer's instructions but using half the amount of each reagent. The use of half of each reagent had an approximately 5% consistent reduction of phagocytosis (Figure S1). The % of polymorphonuclear cells (PMN or granulocytes) was assessed by using side scatter and forward scatter after gating on nucleated cells on FL2 (i.e., DNA staining solution). Percentage phagocytosis on all leukocytes, PMN, and monocytes was determined (Figure S2).

### 2.9. Adipose Biopsy

Subcutaneous adipose tissue was collected by biopsy from alternate sides of the tail-head 1 d before and 7 d after IMI (Figure 1). The biopsy was performed as previously described [38] with modifications. Briefly, the clipped tail-head area was thoroughly scrubbed with poviderm scrub (Povidone Henry Schein Inc., USA) and 2% lidocaine (Henry Schein Inc., USA) was injected subcutaneously in the area of incision. A 4 to 5-cm incision was made to expose the tissue which was collected using sterile forceps and a scalpel. The incision was sutured closed. Biopsied tissue was placed in a sterile Petri dish containing gauze sprayed with RNAse Zap (ThermoFisher, USA). Connective tissue and large vessels were dissected out using a sterile scalpel blade and the tissue was washed using a sterile physiological saline solution with the aid of a disposable sterile syringe. The cleaned tissue was dissected into 3 pieces. Two pieces were transferred to 2.0 mL self-standing cryovials (cat#, 26-201, Genesee Scientific, USA) and put in a foam box with dry ice for transport to the laboratory and then stored at −80°C until analysis. The other piece was put in a 1.5 mL tube containing 10% neutral buffered formalin (#16004-126, VWR, USA) to be fixed for histological analysis.

### 2.10. Mammary Epithelial Cells Isolation

The mammary epithelial cells (MEC) were isolated from 50 mL of milk using magnetic sorting. The milk sample was collected in 50 mL sterile tubes (cat# 89004-364, VWR, USA) and immediately preserved on ice until isolation (approximately 1 h later). Tubes were centrifuged at 1,000 ×g at 4°C for 10 min to separate the fat and pellet the cells. Cells were washed twice with 10 mL of sterile PBS and centrifuged at 500 ×g at 4°C for 5 min. Before the last wash the cells were counted using a MOXI Z Mini Automated Cell Counter (Orflo Technologies, USA). The final pellet was resuspended in 500 *μ*L of a PBS solution plus 0.1% bovine albumin and transferred in a 1.5 mL tube prewetted with the PBS + 0.1% albumin solution. An antibody against the epithelial-specific marker mucin 1 (NBP1-60046, Novus Biologicals, USA) [39] conjugated with Pierce Protein AG Magnetic Beads (ThermoFisher, USA) was added (1 *μ*L/10^6^ cells). Samples were incubated for 30 min on ice in a shaker and cells were washed once using PBS and immediately isolated using an autoMACS separator (Miltenyi Biotec, USA). The positive and the negative separated cells were stored at −80°C until RNA extraction. The evaluation of the enrichment of mammary epithelial cells was performed on 5 positive and 5 negative cells from 5 random milk samples measuring expression of casein kappa* (CSN3)* and lactalbumin* (LALBA)* on MUC1 positive and MUC1 negative cells. Overall, the use of MUC1 antibody tended to be enriched for cells expressing higher amount of the mammary-specific genes casein *κ (CSN3)* and lactalbumin* (LALBA)* plus mucin 1* (MUC1)* (Figure S3). Normalization of transcript abundance was performed as described below.

### 2.11. RNA Isolation and Reverse Transcription Quantitative Polymerase Chain Reaction (RT-qPCR)

RNA extraction was performed using RNA Clean & Concentrator™-5 (cat# R1013, Zymo Research, USA) following the vendor protocol. Prior to RNA extraction, the adipose tissue was disrupted using a Bullet Blender Next Advance (Laboratory Instruments, USA). RNA was quantified with a Nanodrop ND-1000 spectrophotometer (NanoDrop Technologies, Wilmington, DE). RNA integrity was assessed using a 2100 Bioanalyzer Instrument (Agilent, USA) by the Center for Genome Research and Biocomputing at Oregon State University. For the MEC the 260/280 ratio was 1.9 ± 0.4 (mean ± SD) and the RNA Integrity Number (RIN) was 4.7 ± 2.1, with a range from 1 to 9.2. For the adipose tissue the 260/280 ratio was 1.8 ± 0.2 (mean ± SD) and the RIN was 4.0 ± 2.0, with a range from 1 to 9.0.

Primers were designed using Primer Express 3 and tested as previously described [40] (Table S1 in File S2) with modifications. Briefly,* Capra hircus* specific sequences were searched in the National Center for Biotechnology Information (NCBI at https://www.ncbi.nlm.nih.gov/) and blasted against the sheep genome on the University of California Santa Cruz Genome Browser (https://genome.ucsc.edu/) in order to determine the exon-exon junctions. Primer-pairs were blasted using NCBI BLASTN tool. Amplicon from each primer-pair was cleaned using DNA Clean & Concentrator-5 (Zymo Research. USA) and sent to the Center for Genome Research and Biocomputing at Oregon State University for sequencing. Results of sequencing are available in Table S2 in File S2.

Despite the low RIN numbers we proceeded with the RT-qPCR analysis (see Results). Six potential internal control genes (ICG) were tested [i.e., glyceraldehyde 3-phosphate dehydrogenase* (GAPDH)*, ribosomal protein S9* (RPS9)*, ubiquitously expressed transcript* (UXT)*, tyrosine 3-monooxygenase/tryptophan 5-monooxygenase activation protein zeta polypeptide* (YWHAZ)*, glucose-6-phosphate dehydrogenase* (G6PD),* and mitochondrial ribosomal protein L39* (MRPL39)*] based on prior publications [40, 41] using geNorm [42]. For adipose tissue the most reliable normalization factor using the above ICG was obtained (*V*-value = 0.23) by using 5 ICG (all the ICG tested except* MRPL39*). For the MEC 3 ICG (*GAPDH*,* RPS9*, and* YWHAZ*; *V*-value = 0.26) were used to calculate the normalization factor. Target transcripts measured were related to lipid synthesis, PPAR*γ* activation, and inflammation. In particular we measured, in both MEC and adipose tissue, the abundance of PPAR*γ* transcript* (PPARG)* and the PPAR*γ* putative targets Lipoprotein Lipase* (LPL)* and Fatty Acid Synthase* (FASN)*. In adipose tissue we also measured transcription of Acetyl-CoA Carboxylase Alpha* (ACACA)*, Sterol Regulatory Element Binding Transcription Factor 1* (SREBF1)*, and Tumor Necrosis Factor alpha* (TNFA)*. For MEC we measured transcription of* MUC1*, Interleukin 8* (IL8)*, and Chemokine (C-C Motif) Ligand 2* (CCL2)*. The RT-qPCR analysis was performed as previously described [40] with some modifications. Briefly, RevertAid (ThermoFisher, USA) was used as reverse transcriptase following manufacturer's indication and Power SYBR Green Master Mix (ThermoFisher, USA) was used for the qPCR. The PCR reaction was performed in a 7900HT (Applied Biosystems, USA) in MicroAmp® Optical 384-Well Reaction Plate (Applied Biosystems, USA). The reaction was as follows: 2 min at 50°C, 10 min at 95°C, and 40 cycles with 15 s at 95°C followed by 1 min at 60°C. A dissociation curve was performed (gradient from 60°C to 95°C) to check for amplicon quality. Final qPCR data were obtained by using a 6-point 2-fold dilution standard curve. The RT-qPCR was performed following MIQE guidelines [43].

### 2.12. Histological Analysis of the Adipose Tissue

Adipose tissue samples preserved in 10% neutral buffered formalin were immersed in 15% and then in 30% diluted sucrose in PBS before cutting in cryostat sections of 15-*μ*m thickness at a temperature of −27°C. Prior to H&E staining, sections were washed in PBS buffer followed by rinse in water for 30 s. Next, sections were incubated with Harris' Hematoxylin (VWR, US, Cat. number 95057-858) solution for 3 min and rinsed afterwards with warm water for 25 s. The sections were submerged in eosin solution and rinsed with PBS for 1 min. Tissue was washed in 80% ethanol for 1 min and finally immersed in xylene for 10 s, mounted in a microscope slide, and imaged with a 10x magnification objective using a Leica DM6000 microscope (Leica Microsystems Inc., IL, USA). The analysis of adipocytes size was performed using CellProfiler 2.1.1 software (http://cellprofiler.org/) [44]. Besides average area of adipocytes, the frequency of various ranges of area and diameter of adipocytes was measured to estimate the formation of new adipocytes (i.e., small size) and formation of large adipocytes, that is, accumulating large amount of stored triglycerides.

### 2.13. Statistical Analysis

Prior to statistical analysis data were checked for outliers using PROC REG of SAS 9.2 (SAS Institute, Inc., Cary, NC, USA). Data with a studentized *t* > 3.0 were removed. Normal distribution was assessed using PROC UNIVARIATE of SAS. Data with a Shapiro-Wilk test of *P* < 0.01 and statistic < 0.90 and a Kolmogorov-Smirnov *P* < 0.01 were considered not normally distributed and were log_2_ or square root transformed prior to statistical analysis. Data were analyzed with the PROC GLIMMIX procedure of SAS 9.2. Fixed effects in the model were TZD (Z), time (T), mastitis (M), and all interactions with goat as random effect. Due to the irregular timeline of each measurement, the covariance structure SP (POW) for repeated measures was used for analysis. Statistical significance and tendencies were declared at *P* ≤ 0.05 and *P* ≤ 0.10, respectively.

In order to account and correct for difference between groups at baseline (for blood parameters and milk yield it was −7 d and for milk composition it was −8 d relative to IMI), a statistical analysis of Z, M, and Z × M was performed for the milk and blood metabolic parameters. If a difference with *P* < 0.2 in any of the parameters analyzed was observed, data for each sample were corrected arithmetically to obtain the same average between groups at baseline. This was performed by subtracting to each sample the difference between the average at baseline between the CTRL group and the sample's group (i.e., all data were corrected by the CTRL group at baseline). This approach was chosen instead of the classical covariate model at baseline in order to have the average-corrected LSmeans data with standard error at baseline for each group. The corrected dataset was used for the statistical analysis.

The statistical analysis of overall mean adipocyte size was performed using the above model. In order to evaluate the effect in each range of adipocyte areas or diameters, the statistical analysis using the above model was performed for each range. When range and time were combined, the model included Z, M, T, and range and all interactions as main effects.

## 3. Results

Two animals in the MTZD group were removed from the study because they ceased to produce milk after IMI. For this reason we had only *n* = 4 for the MTZD groups. The effect however was not due to an interaction between TZD injection and IMI because, in the follow-up study performed, 1 out of 4 animals in the control group ceased completely milk production after IMI while all of the TZD-treated animals continued to milk. The reason for agalactia in the two animals in TZD group in the main study remains unclear. Five days after IMI a few goats had issues with the catheter (either the catheter was dislodged or was chewed by other goats) and it needed to be removed. In order to keep all the treatments consistent we removed the catheter from all the animals and we injected the 2,4-TZD directly into the jugular vein until the end of the trial.

### 3.1. Rectal Temperature and Body Weight

**Figure 2:**
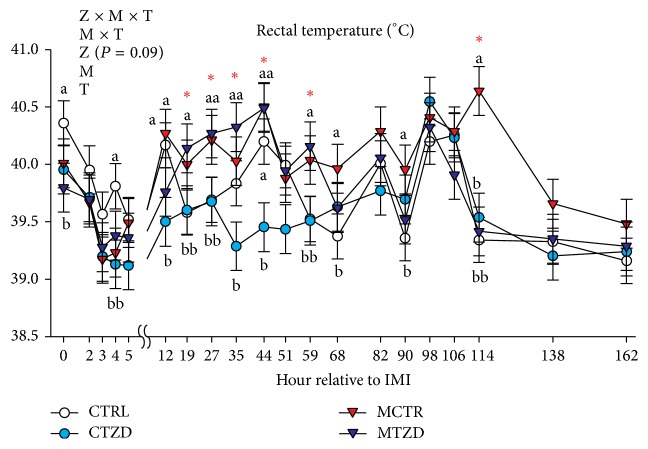
Rectal temperature recorded for several hours after intramammary infusion (IMI) of* Strep. uberis* (M) or saline in goats receiving daily intrajugular injection of 2,4-thiazolidinedione (TZD) or saline (CTR). Significant (*P* ≤ 0.05) effects and interactions are indicated in the graph (mastitis: M, time: T, and TZD: Z). The *P* value for tendency of TZD effect is reported. Significant effect of M × T is denoted with red asterisk and different letters denote significant differences. Letters denoted significant differences between groups at the same time point.

The rectal temperature overall declined in the first 5 hours after IMI in all groups but increased significantly afterwards until 44 hours after IMI, especially in the animals treated with* Strep. uberis* (Figure 2). The rectal temperature tended to remain higher in the MCTR group compared to the other groups until it reached a significantly higher value compared to all the other groups at 114 h (approx. 5 d) after IMI (Figure 2). Temperatures in the MTZD group were similar to the CTRL and CTZD groups within 68 h after IMI. Overall the CTZD group had a more stable and lower temperature compared to all the other groups, including CTRL, especially at 44 h after IMI where a significant increase in body temperature was observed for the CTRL animals. Body weight was not affected by TZD or mastitis (Figure S4).

### 3.2. Milk Yield and Composition

**Figure 3:**
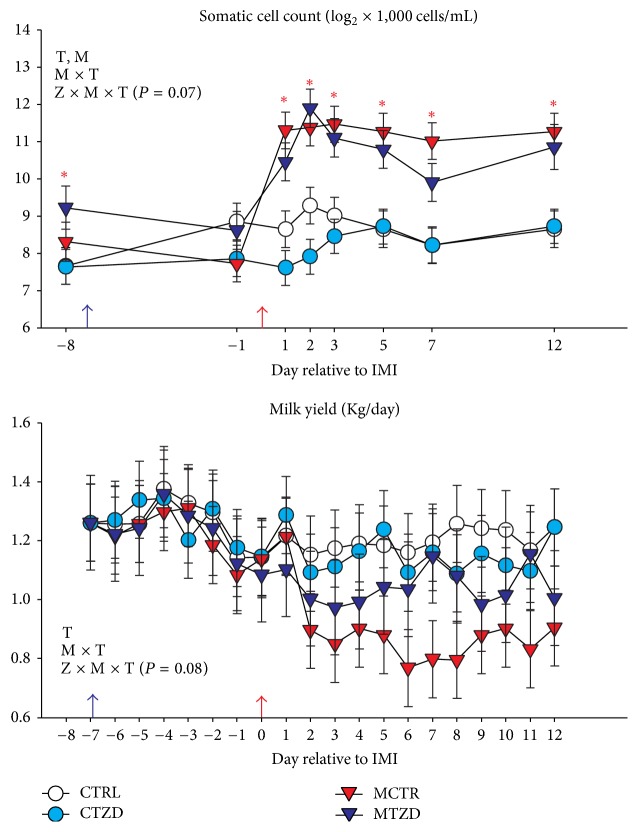
Milk somatic cell count and milk yield measurements from −8 to 12 d relative to intramammary infusion of* Strep. uberis* (M) or saline in goats receiving daily intrajugular injection of 2,4-thiazolidinedione (TZD) or saline (CTR). Significant (*P* ≤ 0.05) effects and interactions are indicated in the graph (mastitis, M, time: T, and TZD, Z). The *P* value for tendency is reported. Significant effect of M × T is denoted with red asterisk.

In Figure 3 is depicted the trend for SCC and milk yield. SCC was overall significantly higher in the groups receiving* Strep. uberis* (i.e., MTZD and MCTR) compared to the groups receiving intramammary infusion of saline at the beginning of the trial, but the differences disappeared just before IMI. Due to IMI, the MTZD and MCTR groups had a significantly larger increase in SCC (13.9- and 11.3-fold, resp., from −1 to 2 days after IMI) than the control groups (1.3 for CTRL and 1-fold for CTZD). The SCC remained higher in MCTR and MTZD compared to CTRL and CTZD until the end of the trial. A tendency for a Z × M × T was detected due to an overall lower SCC for the animals receiving TZD, especially for the CTZD group during the first 2 days after IMI.

Despite the significant difference observed at −8 d, which would have prompted us to correct the data based on the criteria utilized in the present work, we decided to show the original SCC data due to the practical importance of the SCC data for farmers; however, when the data were adjusted at −8 d, the MCTR had an overall larger SCC compared to all groups, including MTZD (Figure S5).

The milk yield at baseline was 1.12 ± 0.35 kg/d. A significant effect of M × T was detected due to an overall decrease in milk yield by the goats treated with* Strep. uberis* compared to goats receiving intramammary saline (Figure 3). A tendency for the interaction Z × M × T (*P* = 0.08) was detected for milk yield.* Strep. uberis* intramammary infusion decreased milk yield in the MCTR group, while the MTZD had only a numerical decrease in milk yield.

**Figure 4:**
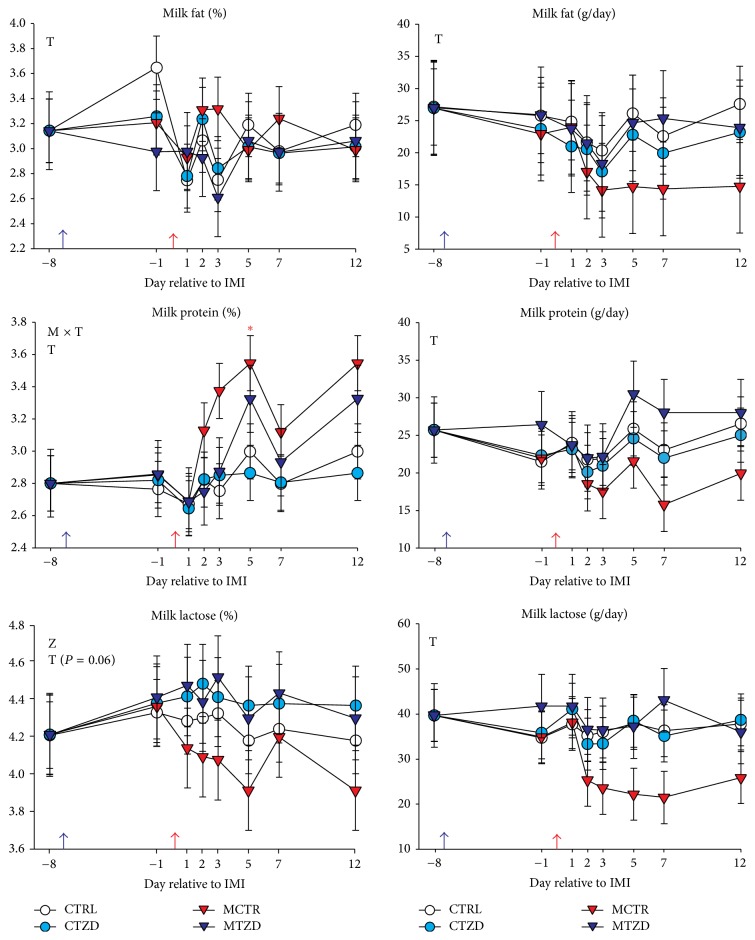
Milk components from −8 to 12 d relative to intramammary infusion of* Strep. uberis* (M) or saline in goats receiving daily intrajugular injection of 2,4-thiazolidinedione (TZD) or saline (CTR). Significant (*P* ≤ 0.05) effects and interactions are indicated in the graph (mastitis: M, time: T, and TZD: Z). The *P* value for tendency is reported. Significant effect of M × T is denoted with red asterisk.

The other milk parameters (Figure 4) were not affected by IMI or TZD except % protein, which was higher in* Strep. uberis* treated goats after IMI likely driven by the higher SCC, and % of milk lactose, which was higher for TZD-treated animals after IMI. Even though not statistically significant, the milk fat yield was numerically lower in MCTR after IMI. The ECM was not significantly affected by treatments but a tendency for M × Z × T was detected due to a decrease in ECM in MCTR but not in the other groups (Figure S6).

### 3.3. Blood Metabolic Parameters

**Figure 5:**
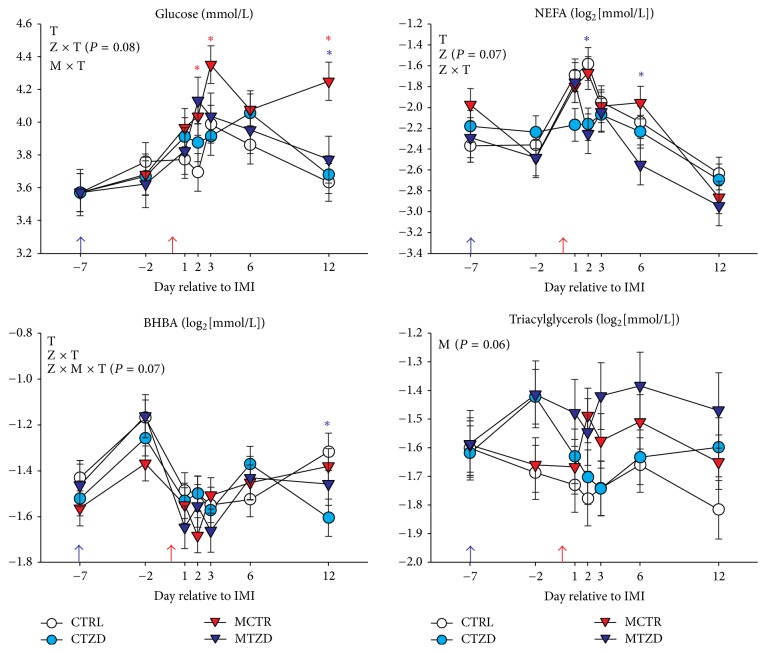
Plasma concentration of blood metabolic parameters from −7 to 12 d relative to intramammary infusion of* Strep. uberis* (M) or saline in goats receiving daily intrajugular injection of 2,4-thiazolidinedione (TZD) or saline (CTR). Significant (*P* ≤ 0.05) effects and interactions are indicated in the graph (mastitis: M, time: T, and TZD: Z). The *P* value for tendency is reported. Significant effect of M × T is denoted with red asterisk and effect of Z × T is denoted with purple asterisk.

Glucose concentration was not affected by TZD but overall higher glucose was detected after IMI in animals receiving* Strep. uberis*, mostly due to a large increase in glycaemia in MCTR group whereas the MTZD group did not have any difference from the nonmastitis groups (Figure 5). We observed an overall increase in NEFA due to IMI in all groups except CTZD (Figure 5). The non-TZD-treated animals had a more persistent increase in NEFA after IMI compared to TZD-treated animals. Overall, NEFA tended to be lower in TZD-treated animals. All animals had a decrease in BHBA after IMI and an overall lower value for TZD was observed at 12 d after IMI (Figure 5). The concentration of triacylglycerol (TG) was not affected by Z or Z × T but tended to be higher (*P* = 0.06) in* Strep. uberus* treated animals after IMI (Figure 5).

A trend (*P* = 0.09) for TZD, mastitis, and full interaction Z × M × T resulted in the MTZD group with a greater urea concentration than the other groups on d 1, 6, and 12 after IMI (Figure S7). After IMI in all groups *α*-tocopherol decreased significantly with no differences between groups (Figure S7).

Administration of TZD had no effect on plasma insulin concentrations, QUICKI, or RQUICKI, although a numerically larger QUICKI was detected for TZD-treated versus control goats (Figure S8). The statistically significant effect of mastitis on insulin was also observed prior to IMI; thus, it cannot be considered biologically relevant (Figure S8).

### 3.4. Blood Minerals

**Figure 6:**
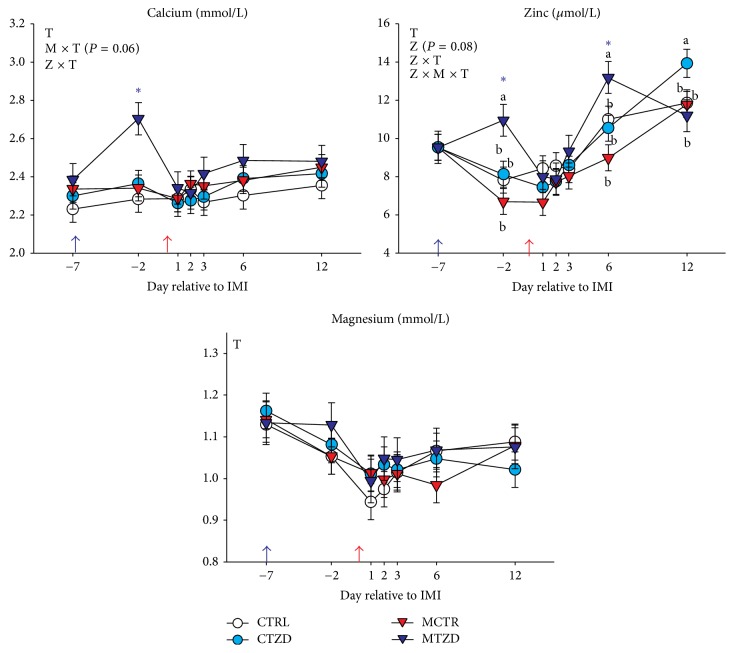
Plasma concentration of blood minerals from −7 to 12 d relative to intramammary infusion of* Strep. uberis* (M) or saline in goats receiving daily intrajugular injection of 2,4-thiazolidinedione (TZD) or saline (CTR). Significant (*P* ≤ 0.05) effects and interactions are indicated in the graph (mastitis: M, time: T, and TZD: Z). The *P* value for tendency is reported. Significant effect Z × T is denoted with purple asterisk.

The Ca was increased in TZD-treated goats prior to IMI and tended to remain higher compared to the non-TZD-treated goats after IMI mainly due to higher Ca concentration in MTZD-treated goats (Figure 6). The concentration of Zn tended (*P* = 0.08) to be higher in TZD-treated compared to non-TZD-treated goats and was overall higher in the former compared to the latter after IMI (Figure 6). Zn concentration was larger in MTZD compared to the other groups at d −2 and 6 relative to IMI but the Zn was higher in CTZD animals at 12 d after IMI. As for Ca, the observed pattern of Zn in blood was mostly driven by MTZD group. The Mg level decreased after IMI but no differences between groups were detected (Figure 6).

### 3.5. Inflammation and Liver Stress

**Figure 7:**
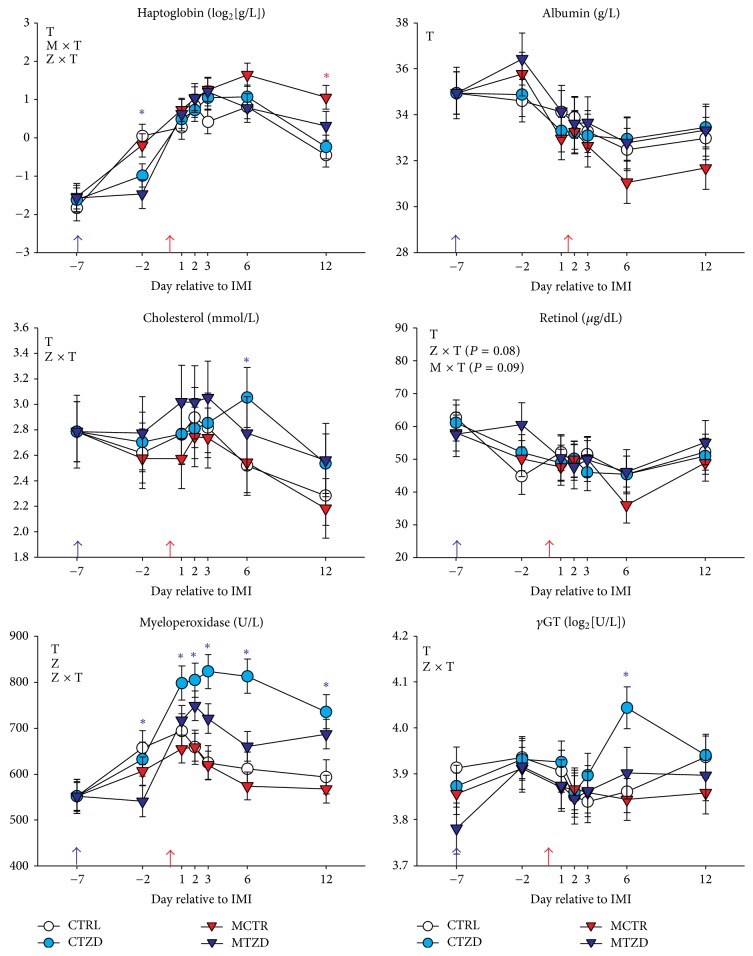
Plasma concentration of blood inflammatory markers and liver stress, including the positive acute phase protein haptoglobin, the markers of negative acute phase albumin, total cholesterol, and retinol, the neutrophils killing capacity marker myeloperoxidase, and the marker of liver stress/disease gamma-glutamyl transferase (*γ*GT) from −7 to 12 d of* Strep. uberis* (M) or saline intramammary infusion (IMI) in goats receiving daily intrajugular injection of 2,4-thiazolidinedione (TZD) or saline (CTR). Significant (*P* ≤ 0.05) effects and interactions are indicated in the graph (mastitis: M, time: T, and TZD: Z). The *P* value for tendency is reported. Significant effect of M × T is denoted with red asterisk and effect of Z × T is denoted with purple asterisk.

The level of the positive acute phase protein (+APP) haptoglobin was 0.30 ± 0.25 g/L (mean ± SD) seven days prior to IMI with a significantly lower overall level in TZD-treated versus control animals after 1 week of TZD treatment (Figure 7). Haptoglobin level rose rapidly after IMI in all animals, reaching values >2 g/L with an overall larger persistence of high levels in* Strep. uberis* treated animals, mostly due to MCTR group (Figure 7). The other measured +APP, ceruloplasmin, was increased over time after IMI in all groups without being significantly affected by TZD or IMI but with numerically lower values in TZD-treated goats versus control at the end of the trial (Figure S9).

The marker of neutrophils killing capacity myeloperoxidase was overall lower in TZD-treated compared to control goats after 1 week of TZD treatment but increased significantly in TZD-treated goats after IMI and was higher compared to control groups until the end of the experiment (Figure 7). The CTZD had an overall higher myeloperoxidase compared to the other groups. The liver stress marker *γ*GT was not different between groups prior to IMI but was overall higher in TZD-treated versus control animals after IMI. The post-IMI difference was mostly due to an increase of the parameter in CTZD 6 d after IMI (Figure 7).

Among the indexes of negative acute phase reaction, albumin was only affected by time with numerically higher values in TZD-treated goats after IMI whereas total cholesterol decreased after IMI in non-TZD-treated groups with significantly lower values compared to TZD-treated goats at 6 d after IMI (Figure 7). Retinol tended to be affected by M × T and Z × T interactions mostly due to higher values in MTZD at −2 d day relative to IMI and lower values in MCTR at 6 d after IMI compared to the other groups (Figure 7). In contrast, the activity of the negative acute phase protein and antioxidant paraoxonase and the total bilirubin, as an index of liver clearance capability, decreased in all groups after IMI without being affected by TZD treatment (Figure S9). The total reactive oxygen metabolites increased in all goats after IMI without being affected by TZD (Figure S9).

### 3.6. % PMN and Phagocytosis in Blood

**Figure 8:**
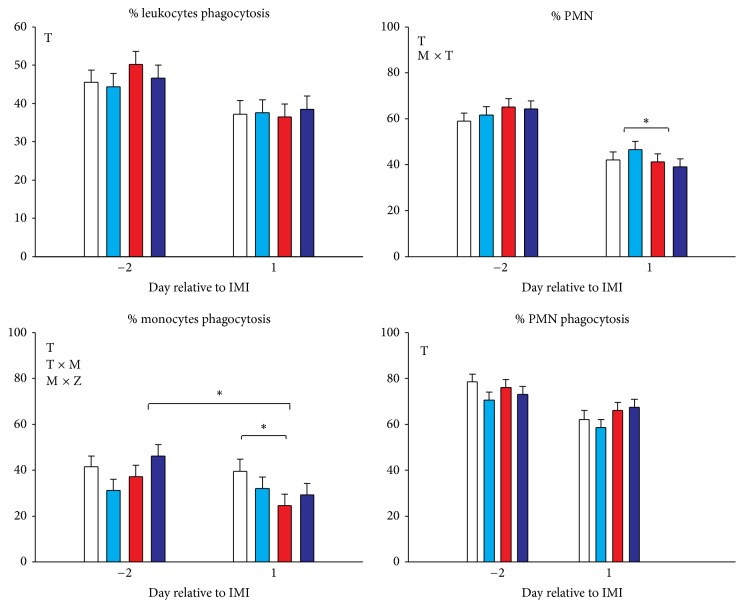
Percentage phagocytosis in leukocytes, polymorphonuclear cells (PMN), and monocytes, and % PMN on all leukocytes in goats receiving intramammary infusion (IMI) of* Strep. uberis* (M) or saline plus daily intrajugular injection of 2,4-thiazolidinedione (TZD) or saline (CTR). Significant (*P* ≤ 0.05) effects and interactions are indicated in the graph (mastitis: M, time: T, and TZD: Z). The *P* value for tendency is reported. Significant differences (*P* < 0.05) are denoted by *∗*.

The % PMN was 62.5 ± 1.7 (mean ± SEM) before IMI and was not different between groups. After IMI the % PMN was decreased in all groups (Figure 8). The goats treated with* Strep. uberis* had a larger decrease in the proportion of PMN compared to the goats receiving intramammary saline (Figure 8). The % phagocytosis of all leukocytes and PMN was only affected by time but was not affected by TZD or mastitis while monocyte phagocytosis was reduced by* Strep. uberis* infusion (Figure 8).

### 3.7. Gene Expression

A negative correlation between cycle to threshold (Ct) or cycle to quantity (Cq) values and RIN has been reported in several publications [45, 46]. This is due to the decrease in abundance of integer mRNA in samples with higher degradation (i.e., lower RIN) resulting in higher Ct (or Cq) values. Based on such correlation, a RIN ≥ 5 was suggested as adequate for RT-qPCR analysis [45]. Due to the large proportion of samples with a RIN < 5 in both adipose tissue (60%) and MEC (50%), most of our samples would have been inadequate for RT-qPCR. The reason for such large proportion of samples with a low RIN is unclear, because samples were snapped frozen on dry ice and kept at −80°C during storage and in ice during analyses. Despite the low RIN, we tested if the degradation observed with the RIN affected the RT-qPCR data. We performed correlation analysis between Cq and RIN. Contrary to the data reported in previous publications above cited, there was no overall significant inverse correlation between Cq values and RIN in all measured genes (Figure S10). Only the Cq values of* FASN* were negatively correlated with the RIN (*r* = −0.23; *P* < 0.05). This negative correlation was however balanced by the significant (*r* = 0.29; *P* < 0.05) positive correlation between Cq and RIN in* GAPDH* (Figure S10). Therefore, the lack of correlation is indicative of the absence of effects of RNA degradation determined by RIN on RT-qPCR results in our experiment. These results can be partly explained by the very short sequence amplified by our primers (amplicon size around 100 bp, File S2) which might have prevented the effect of RNA degradation on Cq values. Due to the lack of correlation between RIN and Cq values, we deemed our RT-qPCR results reliable.

**Figure 9:**
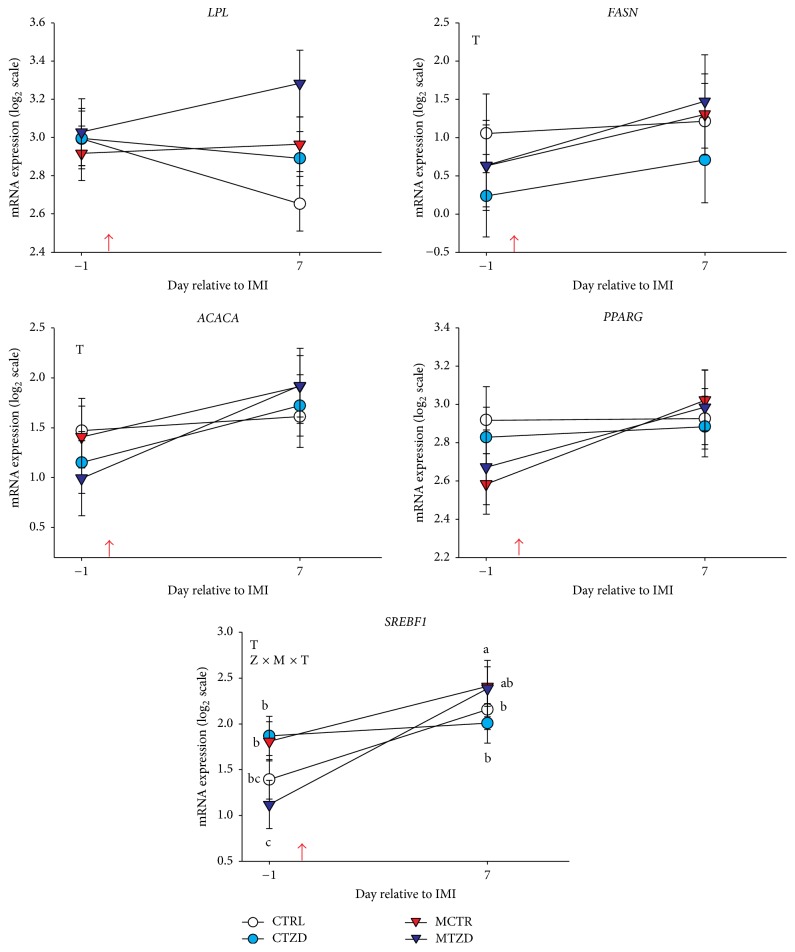
Transcript abundance of selected genes in subcutaneous adipose tissue in goats receiving intramammary infusion (IMI) of* Strep. uberis* (M) or saline plus daily intrajugular injection of 2,4-thiazolidinedione (TZD) or saline (CTR). Significant (*P* ≤ 0.05) effects and interactions are indicated in the graph (mastitis: M, time: T, and TZD: Z).

*Subcutaneous Adipose Tissue*. Results for mRNA expression in adipose tissue are reported in Figure 9. The* TNFA* gene was undetectable in most of the samples so results are not shown. No effects were observed on expression of measured genes by mastitis or TZD treatment with exception of a Z × M × T interaction for* SREBF1* due to a larger overall increase in expression of the gene in MTZD compared to the other groups after IMI. 

**Figure 10:**
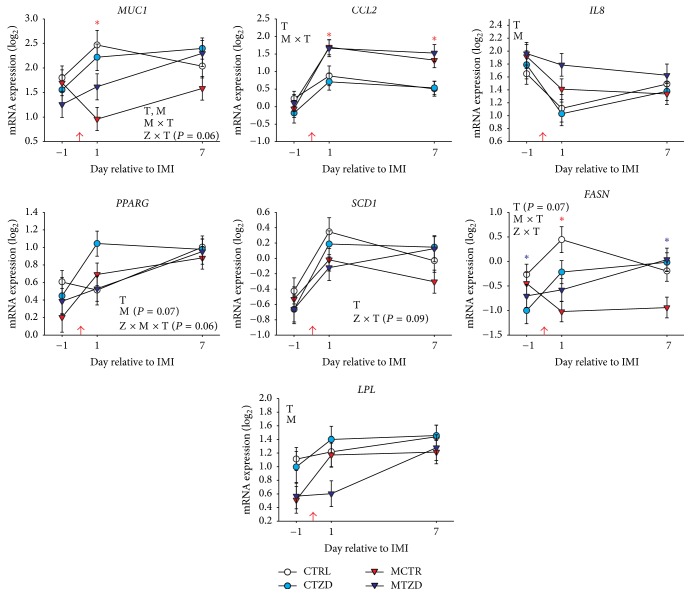
Transcript abundance of selected genes in mammary epithelial cells in goats receiving intramammary infusion (IMI) of* Strep. uberis* (M) or saline plus daily intrajugular injection of 2,4-thiazolidinedione (TZD) or saline (CTR). Significant (*P* ≤ 0.05) effects and interactions are indicated in the graph (mastitis: M, time: T, and TZD: Z). Significant differences due to mastitis × time (M × T) and TZD × time (Z × T) are denoted with red asterisk and purple asterisk, respectively.

*Mammary Epithelial Cells*. Results for the abundance of transcripts in mammary epithelial cells are reported in Figure 10. The expression of* MUC1* was significantly affected by mastitis treatment, with a lower expression in mastitis-treated goats at 1 d after IMI, and a larger expression in TZD-treated versus control goats at 7 d after IMI. Mastitis induction significantly increased the expression of* CCL2* after IMI. Expression of* IL8* decreased over time in all groups but remained higher in goats treated with* Strep. uberis* the day after IMI. The transcript expression of PPAR*γ* and its target genes* SCD1*,* FASN*, and* LPL* all increased or tended to increase through time. TZD-treated goats tended (*P* = 0.08) to have a higher expression of* PPARG* especially at 1 d after IMI. The expression of* SCD1* tended to be higher in TZD-treated goats after IMI. The expression of* FASN* was negatively affected by mastitis, whereas TZD treatment prevented a decrease in expression of* FASN* but delayed the increase in expression of* LPL* in mastitis-treated goats after IMI.

### 3.8. Size of Adipocytes

**Figure 11:**
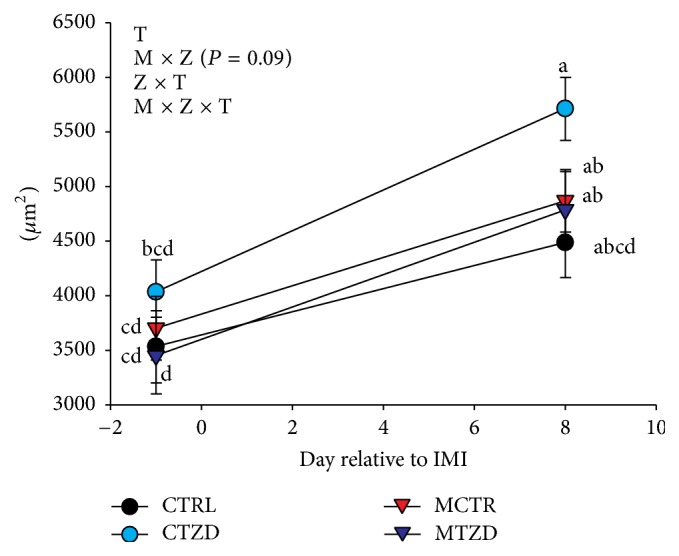
Area of adipocytes in subcutaneous adipose tissue collected from the tail-head of goats receiving intramammary infusion (IMI) of* Strep. uberis* (M) or saline plus daily intrajugular injection of 2,4-thiazolidinedione (TZD) or saline (CTR). Significant (*P* ≤ 0.05) effects and interactions are indicated in the graph (mastitis: M, time: T, and TZD: Z). Significant differences (*P* < 0.05) between time point are denoted by diverse letters.

**Figure 12:**
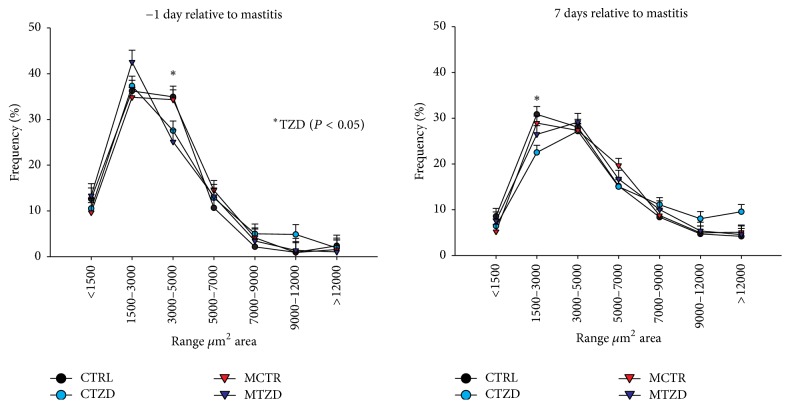
Frequency of adipocytes in subcutaneous adipose tissue collected from the tail-head of goats receiving intramammary infusion (IMI) of* Strep. uberis* (M) or saline plus daily intrajugular injection of 2,4-thiazolidinedione (TZD) or saline (CTR). Significant difference due to TZD is indicated by *∗*.

The average area of adipocytes was significantly increased over time and was affected by M × Z × T because the CTZD group had a larger increase of adipocyte size after IMI compared to any other group (Figure 11). The frequency of adipocytes with medium areas (3,000 to 5,000 *μ*m^2^) was overall lower in TZD versus control goats at 1 day prior to IMI (i.e., 6 days of TZD treatment) and a decrease frequency of small adipocytes (1,500 to 3,000 *μ*m^2^) was more pronounced in TZD-treated goats versus control at 7 days after IMI (Figures 12 and S11). When the diameter of adipocytes was considered (Figures S12 and S13), a lower frequency of medium-size adipocytes (60 to 80 *μ*m) at −1 d relative to IMI was evidenced for TZD versus control animals and a tendency for a higher frequency of large adipocytes (>100 *μ*m) was observed for TZD versus control animals, especially due to CTZD animals.

## 4. Discussion

### 4.1. The Subclinical Mastitis Model Used Works

In the present study all the animals receiving intramammary infusion with* Strep. uberis* developed subclinical mastitis. This was evidenced by the large increase in SCC 24 h after IMI without visible abnormalities in milk. Further support for subclinical mastitis was provided by the significant (although modest) higher rectal temperature between 12 and 44 h after IMI compared to control groups, by the decrease in milk yield, by the increase in expression of* CCL2* in MEC, and by a significant decrease in % PMN in blood within 24 h after IMI, likely caused by a temporary massive migration of neutrophils into the mammary gland. The CCL2 is a chemoattractant for neutrophils and macrophages [47] and crucial for the MEC-macrophages crosstalk [48] and detected to be upregulated in cows receiving IMI with* Strep. uberis* [27]. During inflammatory episodes such as mastitis, plasma concentrations of acute phase proteins such as haptoglobin and ceruloplasmin are likely to increase. Indeed, in the present study all groups had an increase in plasma haptoglobin, indicating that all animals were experiencing some sort of inflammatory conditions. The mean level of haptoglobin before IMI in the goats used in the present experiment (i.e., 0.3 g/L) was higher than what was normally observed in healthy cattle (<0.2 g/L) [33, 49–52] and higher than what was previously observed in healthy goats [53, 54]. A level that is considered high (or animals having a significant acute phase reaction) is when haptoglobin > 0.3 g/L; thus, our animals had some basal level of inflammation. In our experiment, haptoglobin was higher than 0.3 g/L after IMI in all groups and the concentration in plasma of indexes of negative acute phase reaction such as albumin, paraoxonase, total cholesterol, and retinol was decreased, indicating a significant inflammatory state after IMI [49, 51].

Our data confirmed the reliability of the model developed by Lasagno and collaborators [31] that used the same dose of* Strep. uberis* to induce subclinical mastitis in lactating goats. Furthermore, data are indicative of basal inflammatory conditions in the goats used in the present experiment. Positive acute phase proteins are known to be affected by transport and commingling in weaned calves [55]. It might be possible that when we started the experiment (1 week after transport and commingling) the animals were still experiencing some stress and/or inflammatory conditions that have induced slight acute phase response.

### 4.2. Metabolic and Inflammatory Response to Induced Intramammary Infection

Goats receiving intramammary* Strep. uberis* had an increase in plasma glucose and NEFA and decrease in BHBA concentrations after IMI. The pattern of these parameters is consistent with the data reported in a study where induction of mastitis was obtained using the gram negative bacteria* E. coli* in primiparous Holstein dairy cows [56]. Our data are somewhat consistent with the increase in NEFA and glucose in plasma in fed-restricted multiparous dairy cows receiving intramammary* Strep. uberis*, although the increase in NEFA was observed by 12 h but not at 36 h after IMI in that study [57]. In our study the increase in NEFA was also observed in CTRL group, which did not receive intramammary* Strep. uberis* but had a larger rectal temperature compared to CTZD at 12 h and 44 h after IMI. The same group appeared to have had an increase in SCC prior to IMI and had a slightly larger proinflammatory response compared to TZD-treated group. The CTRL group also appeared to have had a similar pattern as the MCTR group for several inflammatory-related parameters, such as albumin, cholesterol, myeloperoxidase, and NEFA, despite not having a significant increase in SCC. On examination of the inflammatory-related parameters, it is apparent that all the animals had an inflammatory response as indicated by a general increase in haptoglobin, ceruloplasmin, and total ROM and a general decrease in magnesium, albumin, cholesterol, and paraoxonase, all markers of inflammatory-like conditions [33, 49, 58]. The change in inflammatory markers was, however, not accompanied by a significant increase in SCC in goats receiving saline in the mammary. The increase in inflammation in goats not receiving* Strep. uberis* could have been partly due to the injection of saline in both halves of the mammary. However physiological saline is known to be relatively innocuous when injected into bovine mammary [59] but stress induced by capturing the animals and frequent injections and samplings can be partly responsible for the observed apparent inflammatory response [60].

Contrary to our findings, there was no decrease in BHBA in the study from Moyes et al. [57] but our data were more similar to the data reported by Graugnard et al. [61], in which IMI with endotoxin was performed in dairy cows. In our study the decrease in BHBA after IMI was observed in all animals, which might be indicative of either an overall response to inflammation (see above) or a metabolic change in all animals unrelated to IMI, likely due to a reduction of the feed intake which reduce the component of BHBA from the rumen fermentation [62]. The increase in glucose in our study can be associated with the decrease in milk yield and, especially, lactose yield, as demonstrated by Moyes and collaborators [56]. All the above observations are indicative of a similar metabolic response among cows and goats to IMI using* Strep. uberis*.

### 4.3. Higher TG, NEFA, and Glucose in Blood Might Indicate an Effect on Insulin Sensitivity by Mastitis

In the study by Moyes and collaborators [57], the cows feed-restricted had an increase in TG after IMI, similar to the tendency we observed in our study. The increase in TG after IMI is not explained by the decrease in uptake of fat by the mammary gland, because a decrease in milk fat synthesis, although only numerically, was observed only for MCTR group, nor can it be explained by a very unlikely increase in feed intake or larger NEFA, which was also present in CTRL group that did not experience an increase in TG after IMI. An increase in TG in blood can be caused by insulin resistance [63]; however, the insulin sensitivity indexes did not indicate a significantly greater insulin resistance in goats receiving* Strep. uberis*. Recently, it was argued and somewhat demonstrated in dairy cows that the insulin indexes used in the present study are probably not a good indicator of true insulin sensitivity [64] and a glucose clamp is likely necessary to accurately measure insulin sensitivity. Therefore, it remains possible that, despite the lack of any indication of insulin resistance by the insulin sensitivity indexes, goats with IMI experienced some decrease in insulin sensitivity.

The increase in glucose and NEFA after IMI observed in our and other studies can be associated with the inflammatory response. The proinflammatory cytokine TNF*α* is known to induce temporary insulin resistance in monogastrics [65]. The work from Kushibiki and collaborators [66] demonstrated that this effect is conserved in ruminants; however, a more recent study by Yuan and collaborators [67] did not demonstrate any effect of TNF*α* injection on glucose, NEFA, or insulin in early lactation dairy cows. In our case, we detected an increase in glucose and NEFA during the first 2 d after IMI but at 3 d after IMI NEFA returned to the value prior to IMI. Similarly, insulin 3 d after IMI was similar to before IMI. The measurement of insulin at 3 d after IMI might have missed the temporary insulin resistance. Somewhat similar to our data, Moyes and collaborators [57] did not find any effect on insulin level in blood after IMI in feed-restricted animals. Interestingly, the same authors detected a large increase in blood insulin after IMI in cows having a positive energy balance. Overall, our data do not allow concluding about the effect of IMI of* Strep. uberis* on insulin sensitivity but the response appears similar to feed-restricted animals.

### 4.4. Response to Mastitis Is Affected by Precondition of the Animals

The observed increase in NEFA after IMI could be a response to lower feed intake, as high plasma NEFA concentrations are indicative of negative energy balance [68]. We did not measure feed intake, but none of the other data, including body weight and BHBA, are indicative of a significant and/or prolonged reduction of feed intake. In addition, the goats were producing a relatively small amount of milk (approx. 1 kg/d), the adipocyte size increased over time, and they were in late lactation; thus, a negative energy balance, if present, was temporary and of little importance. However, as discussed above, the response to mastitis in the present study is somewhat similar to dairy cows in negative energy balance in the study of Moyes et al. [57] and the body condition score of the goats in our study was 1.6 ± 0.5 (mean ± SD; scale 1–5), which is in the low range compared to the values reported in literature for this breed of goats and stage of lactation [69]. In our experiment, the ration was not optimized for lactating goats. This was done with the purpose of being consistent with the diet originally provided by the commercial farm where the goats were purchased. The diet may have not been adequate, as suggested by a response to mastitis more similar to animals in negative energy balance, indicating a possible nutritional deficiency. The possible deficiency could have been partly determined also by the original condition of the animals indicated by the less than optimal body condition and the relatively high acute phase that could have influenced the response to intramammary infection.

### 4.5. Effect of TZD Treatment: General Aspects

2,4-Thiazolidinedione is the basic molecular compound for the chemical synthesis of all thiazolidinedione molecules known to be specific and potent PPAR*γ* agonists, including rosiglitazone and pioglitazone [70, 71]. Because of this, 2,4-thiazolidinedione was assumed to be a PPAR*γ* agonist; however, to our knowledge, it has not been proved that 2,4-TZD is a PPAR*γ* agonist. Indirect support for this compound being a PPAR*γ* agonist is coming from prior studies in ruminants. In heifers and dairy cows it was demonstrated that 2,4-thiazolidinedione injection counteracted the insulin resistance after TNF*α* injection [66] and significantly decreased NEFA post-partum in dairy cows [72], respectively. Based on the above evidence, we assumed, in the present study, that 2,4-TZD is a PPAR*γ* agonist.

### 4.6. Larger Adipocytes Size May Indicate Large Insulin Sensitivity in TZD-Treated Animals

The prevention of insulin resistance in heifers treated with TNF*α* was achieved by treatment with TZD by Kushibiki and collaborators [66]. We did not observe any significant effect of TZD on insulin sensitivity indexes, with only numerically greater QUICKI compared to control animals prior to IMI. Lack of effect on insulin sensitivity indexes in our study is in accordance with data in dairy cows from Schoenberg and Overton [73], where the TZD was used before birth, and Yousefi and collaborators [74], where pioglitazone, another thiazolidinedione and a well-established synthetic PPAR*γ* ligand, was used during the entire transition period.

As stated above, the insulin sensitivity indexes used may not apply to ruminants. The larger increase in adipocyte size accompanied by a lower NEFA in CTZD animals after IMI compared to the other groups can indicate an improved insulin sensitivity by TZD. An increase in adipocyte size is consequence of TG accumulation. Adipose tissue accumulates TG under the influence of insulin; thus, the data appear to indicate that adipose tissue in CTZD group had greater insulin sensitivity compared to the other groups. However, we are unable without an insulin or glucose clamp to provide a definitive conclusion about insulin sensitivity in this study.

### 4.7. TZD Treatment Helped to Maintain Milk Yield and Milk Fat Production after IMI

Milk yield was not affected by TZD in normal goats but TZD treatment prevented the decrease of milk yield after IMI with* Strep. uberis*. Administration of TZD during the present trial did not significantly affect milk composition but, as with milk yield, milk fat and lactose were not decreased in MTZD as much as with MCTR. A direct effect of TZD on prevention of milk fat synthesis decline can be inferred by the higher expression in MEC of* FASN* in MTZD versus MCTR at 7 d after IMI and a numerically higher expression of* SCD* at the same time point. The higher expression of* FASN* and the lower expression of* LPL* in MTZD versus MCTR may indicate a different effect of TZD on de novo versus preformed fatty acids in milk after IMI [17]. Interestingly, milk fat depression is characterized by a larger decrease in expression of* FASN* compared to* LPL* [75]. It has been previously observed in vitro that expression of* FASN*, but not* LPL*, is increased upon activation of PPAR*γ* by rosiglitazone [18]. The response of* FASN* to rosiglitazone appears to be consistent among ruminants, as recently reviewed [13]. Overall, those data might indicate some activation of PPAR*γ* in MEC by 2,4-thiazolidinedione.

### 4.8. TZD Treatment Prevented a Large Acute Phase Reaction and Improved Liver Response to IMI

The larger increase of haptoglobin in non-TZD- versus TZD-treated goats before IMI and after IMI, particularly in MCTR goats, and the higher level of total plasma cholesterol in TZD versus control goats are suggestive of improved liver activity by TZD [34, 51]. A better liver status in TZD-treated versus control animals is also supported by the tendency for a higher Zn level in blood [76]. Taking these findings together, there is an indication that the animals treated with TZD had a lower inflammatory status before IMI but, after IMI, the same animals had a faster recovery of the inflammatory status due to a better response by the liver. Thus, the TZD treatment appears to have improved the response to mastitis.

### 4.9. TZD Increased Killing Capacity of Neutrophils

Recruitment and activation of neutrophils is known to be a principal defense mechanism of innate immunity. In addition, during inflammation, neutrophils are the major cell type observed in the mammary gland [77]; thus, their ability to migrate to the site of infection plays a critical role in the inflammatory response. In the present study, the decrease in the % of blood PMN phagocytosis in plasma 1 d after the IMI and the concomitant increase in SCC are suggestive of a chemotactic process of the mammary toward neutrophils in all groups but larger in goats receiving* Strep. uberis*, also supported by higher* IL8* expression in MEC of goats treated with* Strep. uberis* compared to control groups after IMI. Although no difference in phagocytosis of blood PMN was detected between groups, the larger increase in MPO is indicative of a larger microbial killing capacity in TZD-treated versus control goats [78]. Neutrophil granulocytes have a high abundance of the enzyme MPO, constituting the majority of azurophilic granules. The MPO produces the antimicrobial compound hypohalous acid, which can be released into circulation but it is also pivotal for bacteria killing after phagocytosis [78, 79]. The increase in blood MPO can be due to an increase in neutrophil count [80] and/or by an increase of MPO expression by neutrophils. It has been demonstrated that PPAR*γ* controls MPO expression in certain activated macrophages [81] but rosiglitazone decreased MPO content of neutrophils in rabbits [82] and pioglitazone decreased both number of neutrophils and expression of MPO in several species [83]. In our study it is likely that TZD increased the expression of MPO. It is not possible to conclude this with certainty in the present study because we did not perform a whole blood leukocyte count; however, the lack of difference of % PMN between groups supports an increase in release and/or expression of MPO by neutrophils in the TZD group. Higher MPO has been also associated with greater inflammation [84]. Inflammatory markers did not indicate increased inflammation but rather attenuated inflammation in TZD-treated versus control goats; thus, it is more likely that the expression of MPO was increased by TZD. Overall, our data indicate that, despite TZD not having an effect on PMN phagocytosis, the bacteria killing capacity of the neutrophils was likely augmented.

### 4.10. TZD Increased Adipocytes Size Despite Not Having Transcriptomic Effects

Data indicated an effect of TZD on adipose tissue by decreasing the release of NEFA and slightly changing the adipocyte size profile. The histological data indicated that TZD-treated goats had a significant decrease in medium-size adipocytes after 1 week of TZD treatment. An increase of small adipocytes is indicative of active adipogenesis. The formation of new adipocytes generally precedes the formation of large adipocytes [85]. Our data are consistent with this general observation because the proportion of large adipocytes was larger in TZD-treated animals in later stages. PPAR*γ* is a well-established master regulator of adipogenesis [15]. Therefore, it appears that TZD had a biological effect on adipose tissue. Surprisingly, no TZD effect was observed in the expression of target genes in adipose tissue. Lack of effect on expression of genes in adipose tissue is consistent with a study conducted in pregnant dry dairy cows [73]. However, in another study where nonpregnant dry cows were used, the injection of TZD affected the expression of several genes, including PPAR*γ* target genes, but the effect was only temporary despite continuous injection [86].

Despite the absence of a significant effect on expression of several of the main lipogenic genes in adipose tissue, the presence of lower NEFA and larger adipocytes, especially in CTZD group, is indicative of lipid accumulation. An accumulation of triglycerides in adipose tissue can compete for lipid precursors with the mammary gland. This cannot be fully proven in the present work but we can speculate that, if existing, we should have detected a decrease in milk fat production in CTZD animals. We did not observe significant changes in milk fat. However, we did observe a tendency for higher expression in MEC of several lipogenic genes and targets of PPAR*γ* such as* FASN*,* SCD1*, and* PPARG* in CTZD-treated animals [13]. Therefore, we can speculate that TZD in our experiment helped the mammary tissue to maintain milk fat synthesis despite the competition with adipose tissue for fatty acids.

### 4.11. Was the Response to TZD Impaired by Less Than Optimal Body Condition and Dietary Deficiency?

The lack of effect of TZD on expression of PPAR*γ* target genes in adipose tissue and the very small effect on expression of the same genes in MEC are indicative of TZD being a weak activator of PPAR*γ*. However, recent in vitro work performed in bovine mammary cells indicated that in order for 2,4-thiazolidinedione to activate PPAR*γ* the vitamin A metabolite 9-*cis*-retinoic acid is required [11]. The 9-*cis*-retinoic acid is a specific activator of Retinoic-X-Receptor, the obligate heterodimer of PPAR*γ* [13]. As discussed above, the goats in the present experiment appeared to have a response to* Strep. uberis* similar to cows in negative energy balance. Our goats were thin and they came from a farm where they never grazed and received only hay and a small amount of supplement. This feeding regime probably provided an insufficient amount of retinol. It is therefore possible that our goats had some deficiency in vitamin A and, thus, a lower level of 9-*cis*-retinoic acid reducing the response to PPAR*γ* activator. Unfortunately, we did not measure level of 9-*cis*-retinoic acid in plasma in the present experiment.

## 5. Limitations and Opportunities of the Study

The present study presents several limitations. These include the availability of goats with a body condition which was less than optimal and lack of a balanced ration for lactating goats both prior to and during the experiment. Furthermore, we did not measure individual feed intake. The above limitations can be however considered also an opportunity. Dairy cows, in general, experience negative energy balance and deficiency of various nutritional compounds in early postpartum period. This is also the time of higher incidence of mastitis; thus, our model appears to be more relevant for early rather than late lactating cows. The inferred possible deficiency (or low level) of 9-*cis*-retinoic acid in blood could have decreased the response of PPAR*γ* to TZD. For the above reasons, a future experiment considering the same treatment in animals with good body condition and well-fed, including adequate level of vitamin A, should be performed.

The interpretation of the large increase in myeloperoxidase would have been easier with the white blood cell count data. The use of insulin and various insulin sensitivity indexes does not appear to be adequate; the use of an insulinaemic clamp could have aided in data interpretation. The low RIN of our total RNA is less than ideal. Despite this we demonstrated that the low RIN is not always a problem and data can be used if a lack of effect of the RIN on gene expression is demonstrated.

Finally, the study would have benefitted from the use of a larger number of animals. Six animals can provide enough statistical power but, as was in our case, the loss of 2 animals had likely reduced the capability of observing biologically relevant effects.

## 6. Summary and Conclusions

The subclinical mastitis model used in the present experiment was previously established. Here we have confirmed the validity of this model and it can be used for future studies. Furthermore, the response of the goats to intramammary infusion of* Strep. uberis* is somewhat similar to dairy cows experiencing negative energy balance.

Administration of TZD to lactating dairy goats undergoing subclinical mastitis had a relatively modest effect on milk yield and components and on overall metabolism with a possible temporary decrease on insulin sensitivity which was improved by TZD treatment. The TZD injection had a strong effect on the inflammatory response and SCC. Especially noteworthy was the effect of TZD on the liver, with a better overall response to the inflammatory situation after IMI, and on neutrophils functions. Despite not having any effect on leukocyte phagocytosis, TZD increased the killing capacity of neutrophils (i.e., myeloperoxidase). Overall the data indicated that the animals treated with TZD had an improved organismal response to the mammary infection.

The milk fat synthesis was not increased but the decline of milk fat synthesis due to mammary infection was partially attenuated by TZD treatment. Although we detected some biological effects of TZD injection, we did only observe a modest effect on expression of PPAR*γ* target genes in MEC but not effects on adipose tissue. The less than expected response on gene expression is indicative of TZD being a relatively weak activator of PPAR*γ*. It is also possible that, in the present study, the lack of a transcriptomic effect of TZD was due to the less than adequate conditions of the animals and/or some sort of nutritional deficiency. We speculate that the goats in the present experiment had a less than adequate amount of vitamin A and of its metabolite 9-*cis*-retinoic acid. This speculation deserves further investigations.

Our hypotheses were that activation of PPAR*γ* (1) improves the response to mammary infection and (2) augments synthesis of milk fat. We can conclude that TZD injection improved the overall response to mammary infection with no or minimal effect on milk synthesis and gene expression. Our data indicate the possibility that PPAR*γ* was only weakly activated by TZD, impeding a full demonstration of the above hypotheses. In order to fully prove the above hypotheses a study using an adequate diet in goats with a good body condition is required.

## Supplementary Material


**File S1.** Results from the BLAST analysis using NCBI of the sequencing of the DNA isolated form the *Strep. uberis* used in the present experiment.
**File S2**. Tables S1 contains the features of primer-pairs used in the present experiment. Table S2 contains the sequence of the amplicon for each primer-pair used
**Figure S1**. Percentage phagocytosis in 100 μL whole blood obtained from two lactating goats using the recommended (1x reagent) or half (0.5x reagents) of amount of reagents using the Phagotest kit (Glycotope, Germany).
**Figure S2**. Schematic visualization of the steps to determine % phagocytosis in granulocytes (or PMN) and monocytes and differential from whole blood of goat using flow cytometer following the instructions of the Phagotest kit (Glycotope, Germany).
**Figure S3**. Transcript abundance of casein κ (*CSN3*), lactalbumin (*LALBA*), and mucin 1 (*MUC1*) in positively vs. negatively magnetically isolated mammary somatic cells using mucin 1 antibody. Reported are the values of RTqPCR data non-normalized and normalized using three internal control genes for mammary epithelial cells (see materials and methods)
**Figure S4**. Body weight measurements relative to *Strept. uberis* (M) or saline intramammary infusion (IMI) in goats receiving daily intrajugular injection of 2,4-thiazolidinedione (TZD) or saline (CTR).
**Figure S5**. Milk somatic cell count (#cells x 1,000/mL of milk) corrected by CTRL group at -8d relative to intramammary infusion (IMI) of *Strep*.*Uberis* (M) or saline during daily injection of 2,4-thiazolidinedione (TZD) or saline (CTR). Significant difference due to mastitis × time (M × T) and mastitis × TZD (M × Z) are denoted with red **∗** and blue **∗**, respectively.
**Figure S6. **Energy corrected milk in goats receiving intramammary infusion (IMI) of *Strept. uberis* (M) or saline plus daily intrajugular injection of 2,4-thiazolidinedione (TZD) or saline (CTR). Significant (P≤0.05) effects and interactions are indicated in the graph (mastitis = M, Time = T, TZD = Z).
**Figure S7**. Plasma concentration of urea and α-tocopherol in goats receiving intramammary infusion (IMI) of *Strept. uberis* (M) or saline plus daily intrajugular injection of 2,4-thiazolidinedione (TZD) or saline (CTR). Significant (P≤0.05) effects and interactions are indicated in the graph (mastitis = M, Time = T, TZD = Z).
**Figure S8**. Plasma concentration of insulin plus Quantitative Insulin Sensitivity Check Index (QUICKI) and the Revised QUICKI (RQUICKI) in goats receiving intramammary infusion (IMI) of *Strept. uberis* (M) or saline plus daily intrajugular injection of 2,4-thiazolidinedione (TZD) or saline (CTR). Significant (P≤0.05) effects and interactions are indicated in the graph (mastitis = M, Time = T, TZD = Z).
**Figure S9**. Plasma concentration of positive (ceruloplasmin) and negative (paraoxonase) acute phase reaction markers, total bilirubin as index of liver clearance, and total reactive oxygen metabolites (ROM) as index of oxidative stress in goats receiving intramammary infusion (IMI) of *Strept. uberis* (M) or saline plus daily intrajugular injection of 2,4-thiazolidinedione (TZD) or saline (CTR).
**Figure S10. **Pearson correlation between RNA Integrity Number (RIN) as calculated by Bioanalyzer and Ct values obtained by RTqPCR for all measured genes combining results from adipose and mammary epithelial cells. Reported in each graph are the value of the Pearson correlation coefficient r and its statistical significance.
**Figure S11**. Adipocytes area in sub-cutaneous adipose tissue in goats receiving intramammary infusion (IMI) of *Strept. uberis* (M) or saline plus daily intrajugular injection of 2,4-thiazolidinedione (TZD) or saline (CTR) at -1 and 7 day post-IMI. Upper panel is the result of the statistical analysis considering all interactions, including adipose area range. The lower panel represents the analysis of each individual adipose area range for all groups of goats with both time points. Significant (P≤0.05) effects and interactions are indicated in the graph (mastitis = M, Time = T, TZD = Z).
**Figure S12**. Diameter of adipocytes in sub-cutaneous adipose tissue in goats receiving intramammary infusion (IMI) of *Strept. uberis* (M) or saline plus daily intrajugular injection of 2,4-thiazolidinedione (TZD) or saline (CTR) at -1 (upper panel) and 7 (lower panel) day post-IMI. Significant (P≤0.05) effects are indicated in the graph (M = mastitis; Z = TZD) by∗.
**FigureS13**. Adipocytes diameter in sub-cutaneous adipose tissue in goats receiving intramammary infusion (IMI) of *Strept. uberis* (M) or saline plus daily intrajugular injection of 2,4-thiazolidinedione (TZD) or saline (CTR) at -1 and 7 day post-IMI. Upper panel is the result of the statistical analysis considering all interactions, including adipose area range. The lower panel represents the analysis of each individual adipose area range for all groups of goats with both time points. Significant (P≤0.05) effects and interactions are indicated in the graph (mastitis = M, Time = T, TZD = Z).
