# Supplementary material for: 2,4-Thiazolidinedione Treatment Improves the Innate Immune Response in Dairy Goats with Induced Subclinical Mastitis
Source: PPAR Res. 2017 Jun 27;2017:7097450. doi: 10.1155/2017/7097450 (PMC5504968; doi:10.1155/2017/7097450)
Supplement: Supplementary file 1 — File S1. Results from the BLAST analysis using NCBI of the sequencing of the DNA isolated form the Strep. uberis used in the present experiment. File S2. Tables S1 contains the features of primer-pairs used in the present experiment. Table S2 contains the sequence of the amplicon for each primer-pair used Figure S1. Percentage phagocytosis in 100 μL whole blood obtained from two lactating goats using the recommended (1x reagent) or half (0.5x reagents) of amount of reagents using the Phagotest kit (Glycotope, Germany). Figure S2. Schematic visualization of the steps to determine % phagocytosis in granulocytes (or PMN) and monocytes and differential from whole blood of goat using flow cytometer following the instructions of the Phagotest kit (Glycotope, Germany). Figure S3. Transcript abundance of casein κ (CSN3), lactalbumin (LALBA), and mucin 1 (MUC1) in positively vs. negatively magnetically isolated mammary somatic cells using mucin 1 antibody. Reported are the values of RTqPCR data non-normalized and normalized using three internal control genes for mammary epithelial cells (see materials and methods) Figure S4. Body weight measurements relative to Strept. uberis (M) or saline intramammary infusion (IMI) in goats receiving daily intrajugular injection of 2,4-thiazolidinedione (TZD) or saline (CTR). Figure S5. Milk somatic cell count (#cells x 1,000/mL of milk) corrected by CTRL group at -8d relative to intramammary infusion (IMI) of Strep.Uberis (M) or saline during daily injection of 2,4-thiazolidinedione (TZD) or saline (CTR). Significant difference due to mastitis × time (M × T) and mastitis × TZD (M × Z) are denoted with red ∗ and blue ∗, respectively. Figure S6. Energy corrected milk in goats receiving intramammary infusion (IMI) of Strept. uberis (M) or saline plus daily intrajugular injection of 2,4-thiazolidinedione (TZD) or saline (CTR). Significant (P≤0.05) effects and interactions are indicated in the graph (mastitis = M, Time = T, TZD = Z). Figure S7 [file 7097450.f1.docx]

## FILE S1

## Streptococcus uberis 0140J complete genome. Sequence ID: [ref|NC_012004.1|](http://www.ncbi.nlm.nih.gov/nucleotide/222152201?report=genbank&log$=nuclalign&blast_rank=1&RID=PVHBPNAJ01R)Length: 1852352N

| Alignment statistics for match #1 | | | | |
| --- | --- | --- | --- | --- |
| Score | Expect | Identities | Gaps | Strand |
| 1038 bits(562) | 0.0 | 759/855(89%) | 9/855(1%) | Plus/Minus |

Query 1 TCATTTGTCCCACCTTCGACGGCTAGCTCCAAAT-GGTTACTCCACCGGCTTCGGGTGTT 59

|||| | ||||||||| | ||||| ||||| ||| |||||| ||||| |||||||||||

Sbjct 19494 TCATCTATCCCACCTTAGGCGGCTGGCTCCTAATAGGTTACCTCACCGACTTCGGGTGTT 19435

Query 60 ACAAACTCTCGTGGTGTGACGGGCGGTGTGTACAAGACCCGGGAACGTATTCACCGTAGC 119

|||||||||||||||||||||||||||||||||||| ||||||||||||||||||| ||

Sbjct 19434 ACAAACTCTCGTGGTGTGACGGGCGGTGTGTACAAGGCCCGGGAACGTATTCACCGCGGC 19375

Query 120 ATGCTGATCTACGATTACTAGCGATTCCAGCTTCATATAGTCGAGTTGCAGACTACAATC 179

|||||||| ||||||||||||||||| |||||| ||| |||||||||| ||||||||

Sbjct 19374 GTGCTGATCCGCGATTACTAGCGATTCCGACTTCATGTAGGCGAGTTGCAGCCTACAATC 19315

Query 180 CGAACTGAGAACAACTTTATGGGATTTGCTTGACCTC-GCGGTTTCGCTGCCCTTTGTAT 238

|||||||||| ||||| | |||| ||||| | || ||| || || | | |||||

Sbjct 19314 CGAACTGAGATTGGCTTTAAGAGATTAGCTTGCCGTCACCGGCTT-GCGACTCGTTGTAC 19256

Query 239 TGTCCATTGTAGCACGTGTGTAGCCCAAATCATAAGGGGCATGATGATTTGACGTCATCC 298

|||||||||||||||||||||||| |||||||||||||||||||||||||||||||

Sbjct 19255 CAACCATTGTAGCACGTGTGTAGCCCAGGTCATAAGGGGCATGATGATTTGACGTCATCC 19196

Query 299 CCACCTTCCTCCGGTTTGTCACCGGCAGTCAACTTAGAGTGCCCAACTTAATGATGGCAA 358

||||||||||||||||| | |||||||||| ||||||||||||||||||||||||||

Sbjct 19195 CCACCTTCCTCCGGTTTATTACCGGCAGTCTCGCTAGAGTGCCCAACTTAATGATGGCAA 19136

Query 359 CTAAGCTTAAGGGTTGCGCTCGTTGCGGGACTTAACCCAACATCTCACGACACGAGCTGA 418

|||| || ||||||||||||||||||||||||||||||||||||||||||||||||||

Sbjct 19135 CTAACAATAGGGGTTGCGCTCGTTGCGGGACTTAACCCAACATCTCACGACACGAGCTGA 19076

Query 419 CGACAACCATGCACCACCTGTCACTCTGTCCCCCGAAGGGGAAAACTCTATCTCTAGAGG 478

|||||||||||||||||||||||| | | |||||| ||| ||||||||||||||

Sbjct 19075 CGACAACCATGCACCACCTGTCAC-CGATGTACCGAAGT--AAAGCTCTATCTCTAGAGC 19019

Query 479 GGTCAGA-GGATGTCAAGATTTGGTAAGGTTCTTCGCGTTGCTTCGAATTAAACCACATG 537

|| || ||||||||||| |||||||||||||||||||||||||||||||||||||||

Sbjct 19018 GGGCATCGGGATGTCAAGACCTGGTAAGGTTCTTCGCGTTGCTTCGAATTAAACCACATG 18959

Query 538 CTCCACCGCTTGTGCGGGTCCCCGTCAATTCCTTTGAGTTTCAACCTTGCGGTCGTACTC 597

|||||||||||||||||| |||||||||||||||||||||||||||||||||||||||||

Sbjct 18958 CTCCACCGCTTGTGCGGGCCCCCGTCAATTCCTTTGAGTTTCAACCTTGCGGTCGTACTC 18899

Query 598 CCCAGGCGGAGTGCTTAATGCGTTAGCTGCAGCACTAAGGGGCGGAAAGCCCCCTAACAC 657

|||||||||||||||||||||||||||| | |||||||| ||||||| || ||||||

Sbjct 18898 CCCAGGCGGAGTGCTTAATGCGTTAGCTCCGGCACTAAGCCCCGGAAAGGGCC-TAACAC 18840

Query 658 TTAGCACTCATCGTTTACGGCGTGGACTACCAGGGTATCTAATCCTGTTTGATCCCCACG 717

|||||||||||||||||||||||||||||||||||||||||||||||||| ||||||||

Sbjct 18839 CTAGCACTCATCGTTTACGGCGTGGACTACCAGGGTATCTAATCCTGTTTGCTCCCCACG 18780

Query 718 CTTTCGCACATCAGCGTCAGTTACAGACCAGAAAGTCGCCTTCGCCACTGGTGTTCCTCC 777

|||||| | |||||||||||||||||||||| || ||| |||||||| |||||||||||

Sbjct 18779 CTTTCGAGCCTCAGCGTCAGTTACAGACCAGAGAGCCGCTTTCGCCACCGGTGTTCCTCC 18720

Query 778 ATATCTCTGCGCATTTCACCGCTACACATGGAATTCCACTTTTCCTCTTCTGCACTCAAG 837

|||| ||| ||||||||||||||||||||||||||||||| | || ||||||||||||||

Sbjct 18719 ATATATCTACGCATTTCACCGCTACACATGGAATTCCACTCTCCC-CTTCTGCACTCAAG 18661

Query 838 TTTTCCAGTTTCCAA 852

||| ||||||||||

Sbjct 18660 TTTGACAGTTTCCAA 18646

**File s2** Table S1. Features of the primers pairs used to analyze gene expression by RTqPCR.

| Gene | Forward primer (5’-3’) | Reverse primer (5’-3’) | Accession no. | bp^1^ |
| --- | --- | --- | --- | --- |
| *ACACA* | GACCTGCTTCAATCCAAGAGGTT | CAAGCTACCATGCCAATCTCATT | XM_005693156 | 200 |
| *CCL2* | AAATTCAATAAGAAGATCCCCATACAG | CCTAGGATGGTCTTGAAAATCACA | XM_005693218 | 100 |
| *CSN3* | GAGCTGACGGTCACAAGGAAA | CTGGGCACCCAAAAATGG | NM_001285587 | 90 |
| *FASN* | GGTGAACTGTCTCCGGAAAGAG | TCGGGCTTGTCTTGTTCCA | NM_001285629 | 100 |
| *GAPDH* | CCATCTTCCAGGAGCGAGATC | CCAGCCTTCTCCATGGTAGTG | XM_005680968 | 100 |
| *IL8* | CAACGGAAAAGAGGTGTGCTTA | GGATCTTGCTTCTCAGCTCTCTTC | XM_005681749 | 90 |
| *LALBA* | GAATTAACTACTGGTT*GG*CCCATAA | CAGAAAGAGGACAGAAGCAGCAA | NM_001285635 | 90 |
| *LPL* | CTTGGAGATGTGGACCAGCTAGT | GGTACGCCTTACTTGGATTTTCTTC | NM_001285607 | 100 |
| *MRPL39* | CAGTATGAAGTGTCAGCGGTTCA | AATGTGCTCTTAAGTGTGCAGGTAGA | XM_005674737.1 | 100 |
| *MUC1* | TCTTTTCGCATTATAAACCTCCAGTT | GCAAAATCACATCCAAAATGCTT | XM_005677405.1 | 100 |
| *PPARG* | TCCGCTCCGCACTACGA | TGATTGCACTTTGGTACTCTTGGA | NM_001285658.1 | 100 |
| *RPS9* | AGGTCTGGAGGGTCAAATTCAC | CAGGGCATTACCTTCGAACAG | XM_005709411 | 100 |
| *SCD1* | GGCGTTCCAGAATGACGTTT | AAAGCCACGTCGGGAATTG | NM_001285619 | 100 |
| *SREBF1* | ACCGACATAGAAGACATGCTTCAG | GCCTTCAAGTGAGGAGCTCATC | NM_001285755 | 100 |
| *TNFA* | CTCTTCTGCCTGCTGCACTTC | AAGATGACCTGAGTGTCTGAACCA | NM_001286442 | 100 |
| *UXT* | AGCGGGACTTGCAAAAGGT | AGCTTCCTGGAGTCGCTCAA | XM_005700842 | 100 |
| *YWHAZ* | TGGTGATGACAAGAAAGGGATTG | TCTGATAGGATGTGTTGGTTGCA | XM_005689196.1 | 100 |

^1^Amplicon size in base pairs (bp).

Table S2. Sequencing results of PCR amplicon obtained from the primer-pairs used for this experiment.

| Gene | Sequence |
| --- | --- |
| *CCL2* | GAGAGTCACCACCAGCAAGTGTCCCAAAGAAGCTGTGATTTTCAAGACCATCCTAGGA |
| *SCD1* | GGGATCGTGCACNACAAGTTTTCAGAAACGGATGCTGATCCCCACAATTCCCGACGTGGCATTACAG |
| *MUC1* | TTCTGAACCCAACCAGCTACTATCAGGAGCTGAAGAGAAGCATTTTGGATGTGATTTTGCA |
| *PPARG* | CTGACGATGGTTGCAGATTATAAGTATGACCTGAAGCTCCAAGAGTACCAAAGTGCAATCAA |
| *FASN* | CGGCGGATCGGTGCGTCCTGGTGTCTAACCTTAACAGCACGTCCCCCATCCCTGAGATAGCCCCGAAGTCCTTGGAGCTGCAGAAGGTGCTCCAGAGTGACCTGGTGATGAACGTCTACCGTGATGGGGCTTGGGGAGCATTCCGCCACTTCCTACTGGAACAAGACAAGCCCGAA |
| *IL8* | GCGAGTTGTGCAGGCATTTTTGAGAGAGCTGAGAAGCAAGATCC |
| *ACACA* | GGGTCNTAGGCACATACATATATGACATCCCAGAAATGTTTCGGCAGTCCCTGATCAAACTCTGGGAATCTATGTCCTCTCAAGCATTCCTTCCACCGCCCCCTCTGCCTTCAGACATACTGACGTACACTGAGCTCGTGTTGGATGATCAAGGTCAACTGGTTCACATGAACAGGCTTCCAGGAGGAAATGAGATTGGCATGGTAGCTTG |
| *SREBF-1* | AGACGCGACTTACCGGGCCTGTTTGACCCGCCCTACGCTGGGGGTGGAGCAGGGACCACAGACCCTGCCAGTCCCGATGTCAGCTCCCCAGGCAGCCTGTCCCCACCTCCTTCCACGATGAGCTCCTCACTTGAAGGCAAA |
| *LPL* | GGCAGCGTTCGTTCTCTCTTGATTGACTCTCTGTTGAATGAAGAAAATCCAAGTAAGGCGTACAGNG |
| *LALBA* | TGGTGAGAAGCCATAGTACAAACAATGACAGCACAGAATATGGACTCTATCA |
| *CSN3* | GAAATTTTCTAGTGTGACTATCCTGGCATTAACCCTGCCATTTTTGGGTGNCCAGAAA |
| *YWHAZ* | GTTCAGCAGCATACCAAGAAGCTTTTGAATCAGCAAAAAGGAAATGCAACCAACACATCCTATCAGAACCNT |

**SUPPLEMENTARY FIGURES**

**Supplemental files caption**

**File S1.** Results from the BLAST analysis using NCBI of the sequencing of the DNA isolated form the *Strep. uberis* used in the present experiment.

**File S2**. Tables S1 contains the features of primer-pairs used in the present experiment. Table S2 contains the sequence of the amplicon for each primer-pair used

**
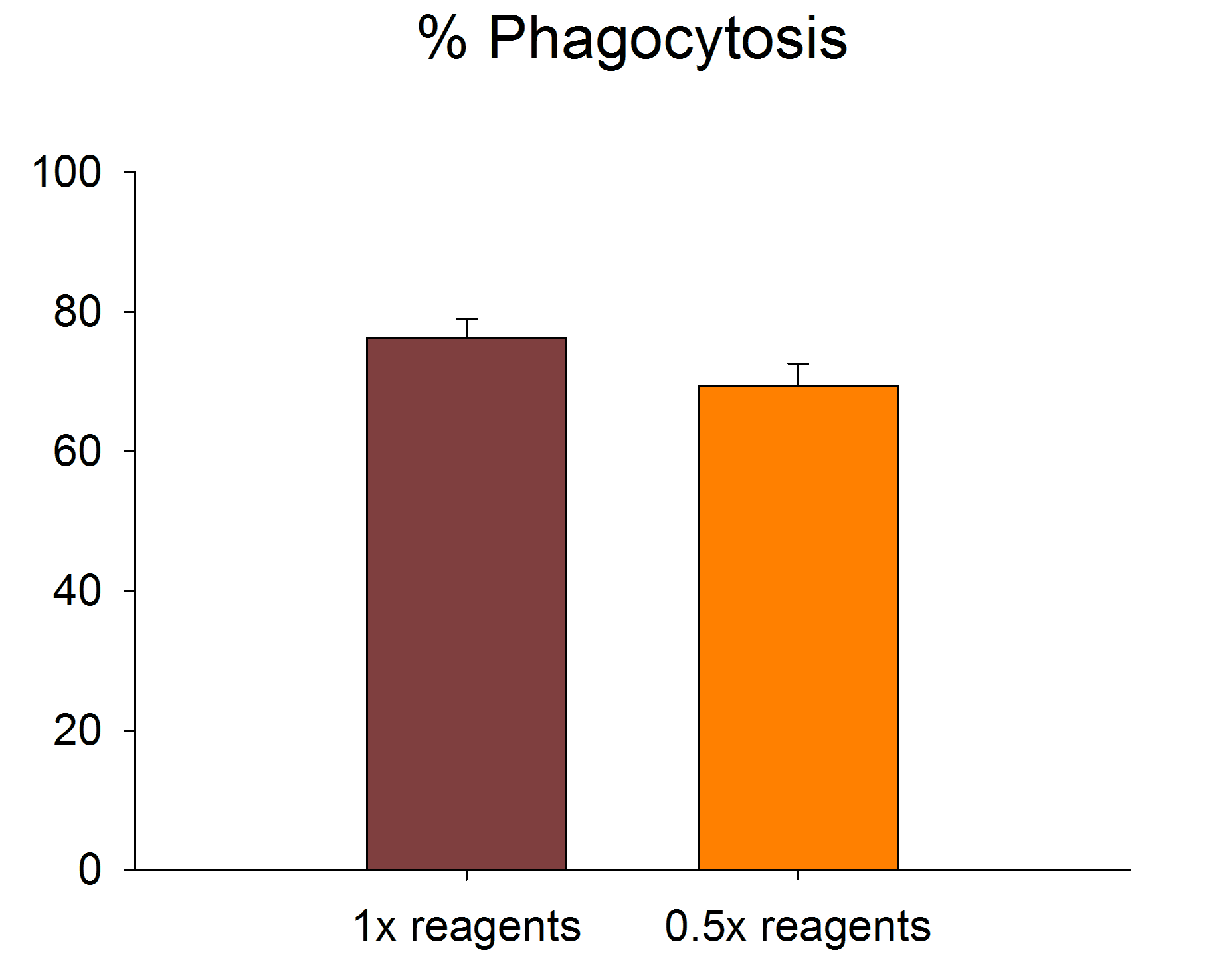
**

**Figure S1**. Percentage phagocytosis in 100 μL whole blood obtained from two lactating goats using the recommended (1x reagent) or half (0.5x reagents) of amount of reagents using the Phagotest kit (Glycotope, Germany).

**
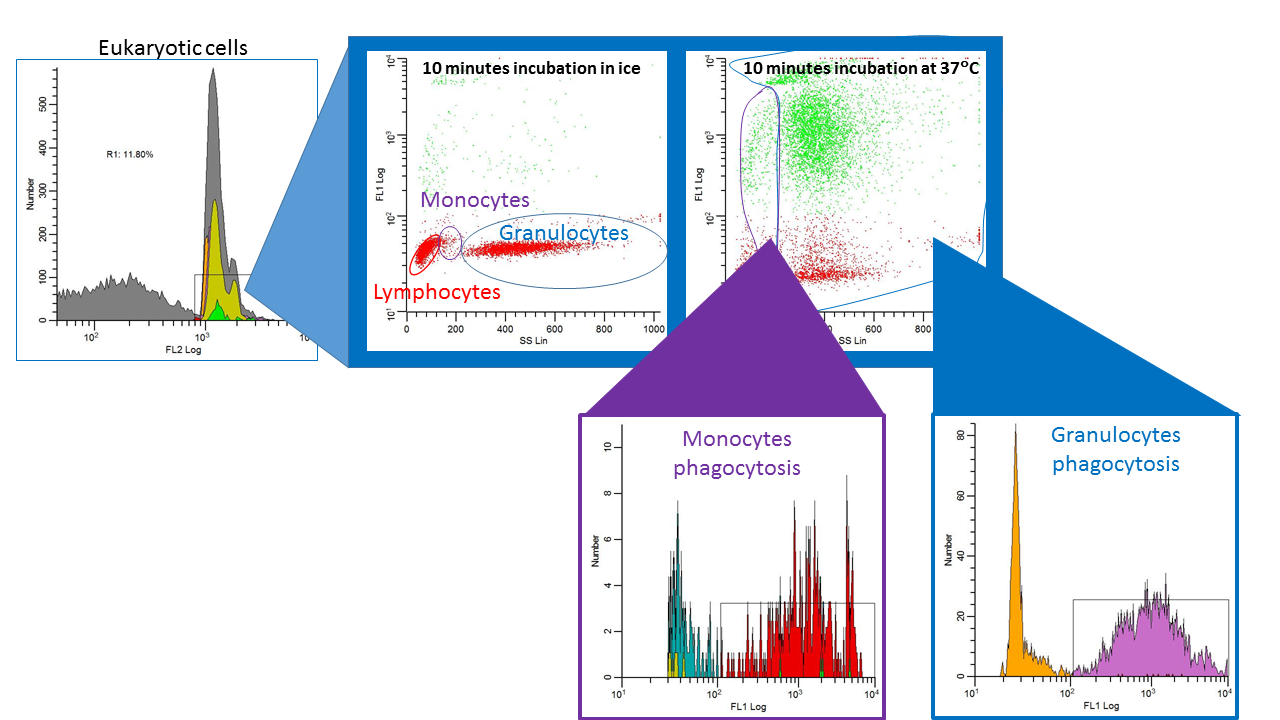
**

**Figure S2**. Schematic visualization of the steps to determine % phagocytosis in granulocytes (or PMN) and monocytes and differential from whole blood of goat using flow cytometer following the instructions of the Phagotest kit (Glycotope, Germany).

**
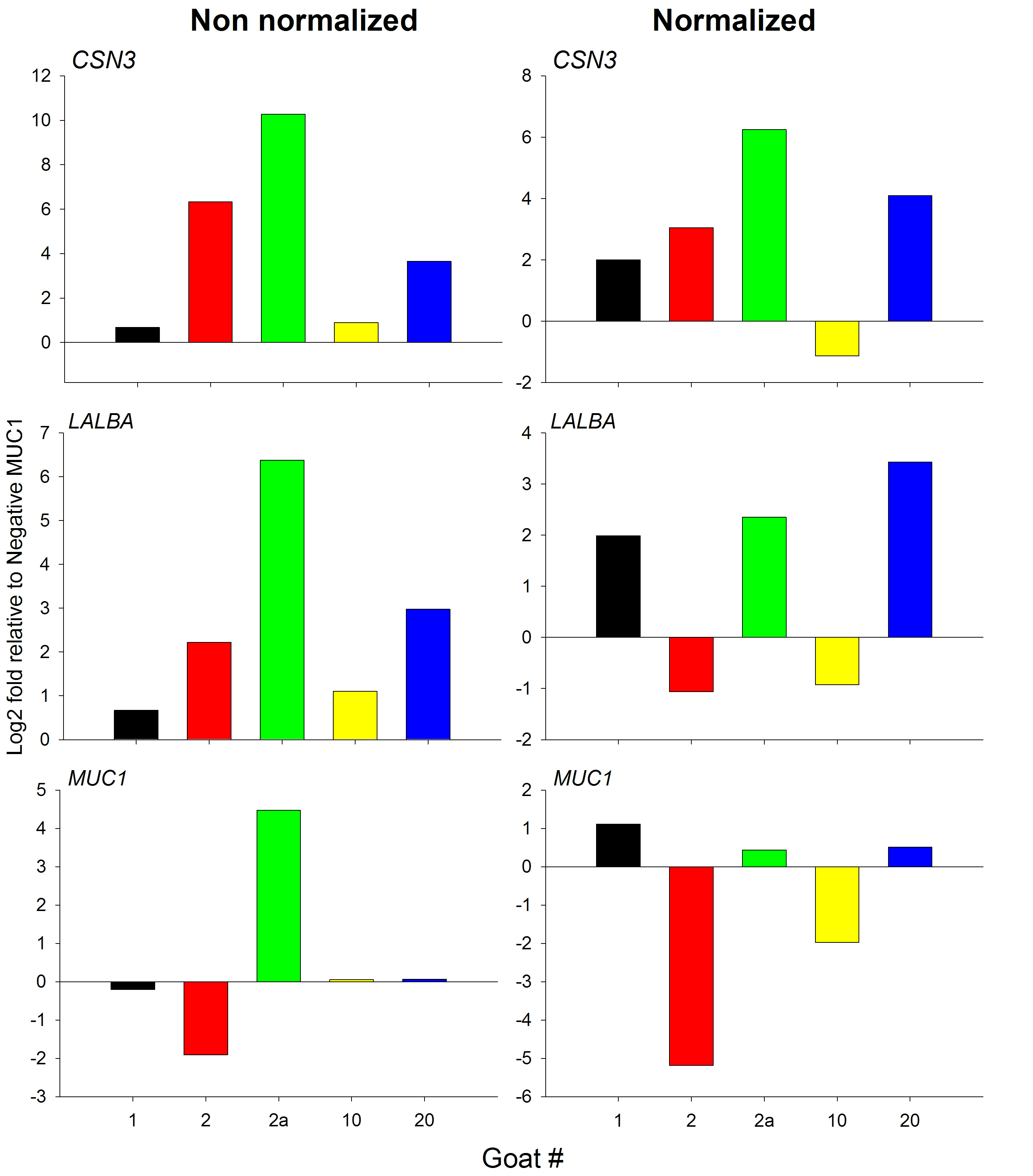
**

**Figure S3**. Transcript abundance of casein κ (*CSN3*), lactalbumin (*LALBA*), and mucin 1 (*MUC1*) in positively vs. negatively magnetically isolated mammary somatic cells using mucin 1 antibody. Reported are the values of RTqPCR data non-normalized and normalized using three internal control genes for mammary epithelial cells (see materials and methods)


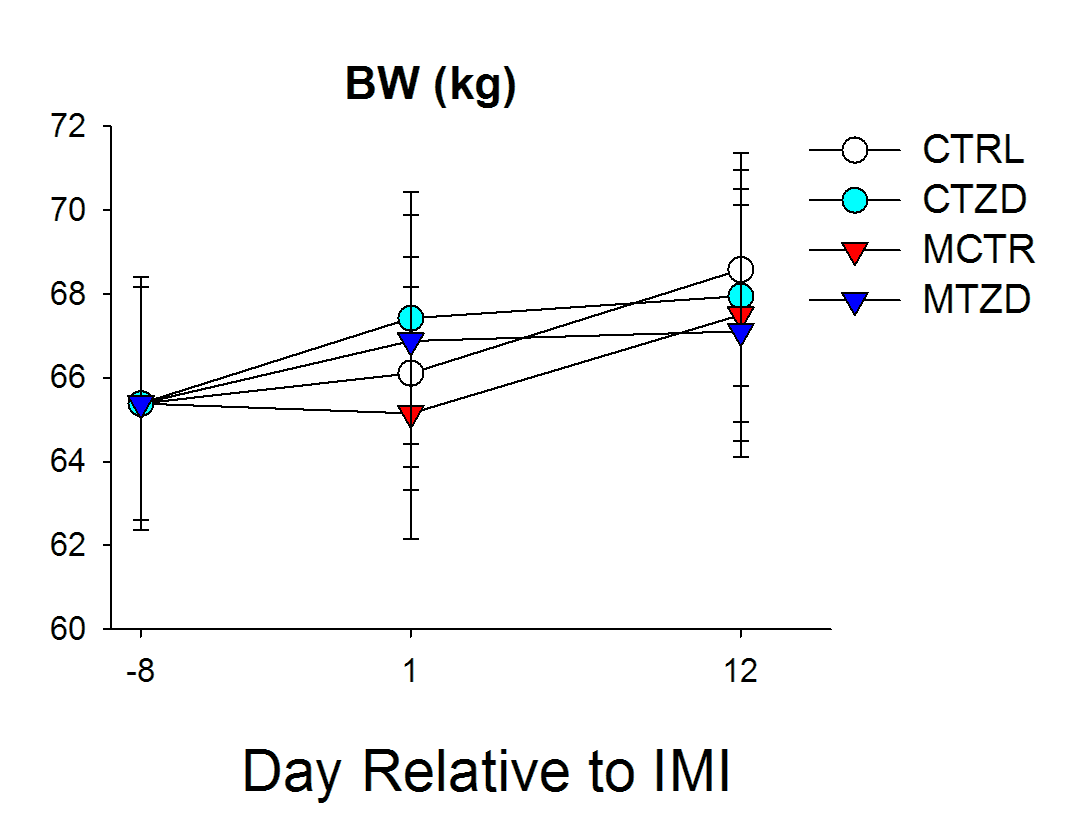


**Figure S4**. Body weight measurements relative to *Strept. uberis* (M) or saline intramammary infusion (IMI) in goats receiving daily intrajugular injection of 2,4-thiazolidinedione (TZD) or saline (CTR).


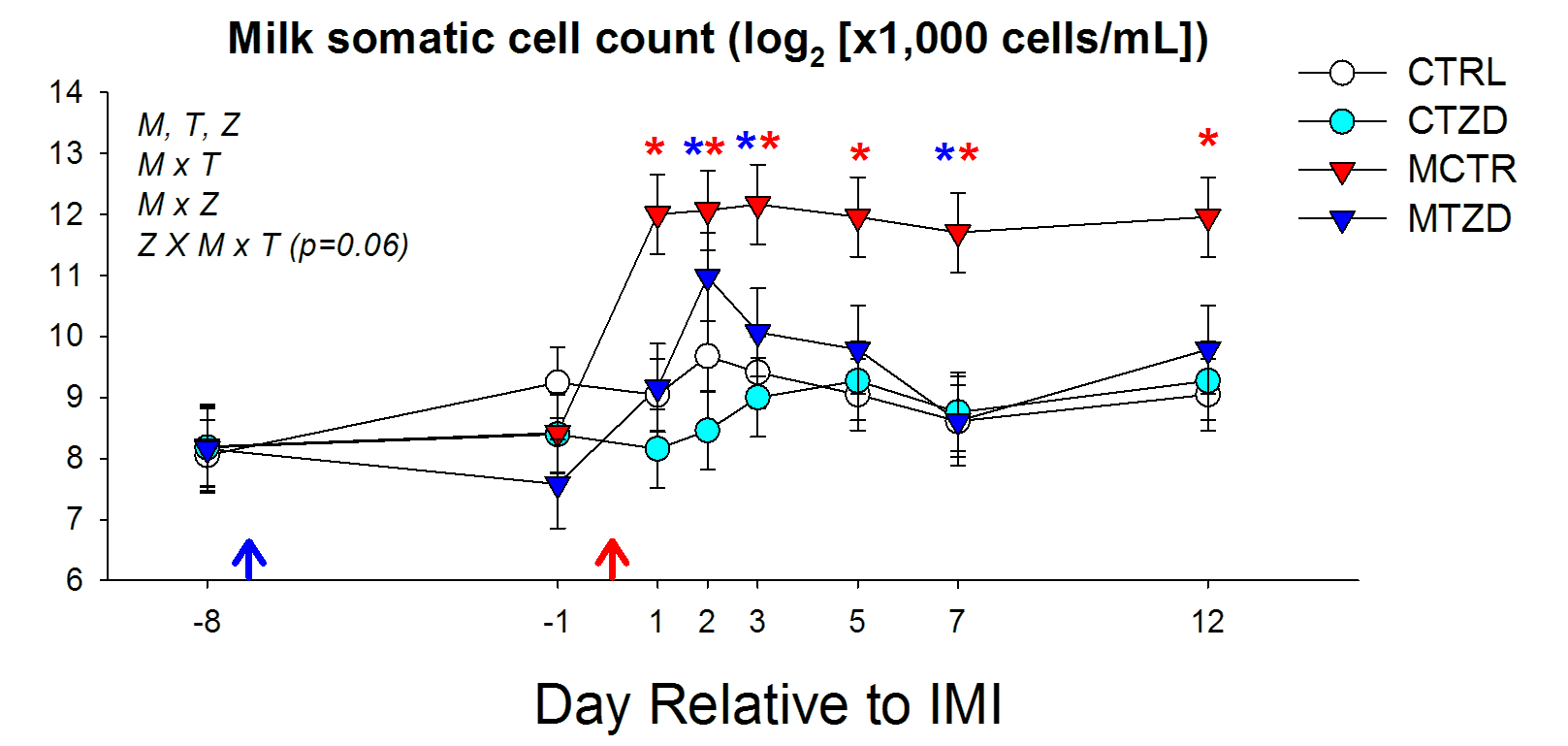


**Figure S5**. Milk somatic cell count (#cells x 1,000/mL of milk) corrected by CTRL group at -8d relative to intramammary infusion (IMI) of *Strep. Uberis* (M) or saline during daily injection of 2,4-thiazolidinedione (TZD) or saline (CTR). Significant difference due to mastitis × time (M × T) and mastitis × TZD (M × Z) are denoted with ***** and *****, respectively.


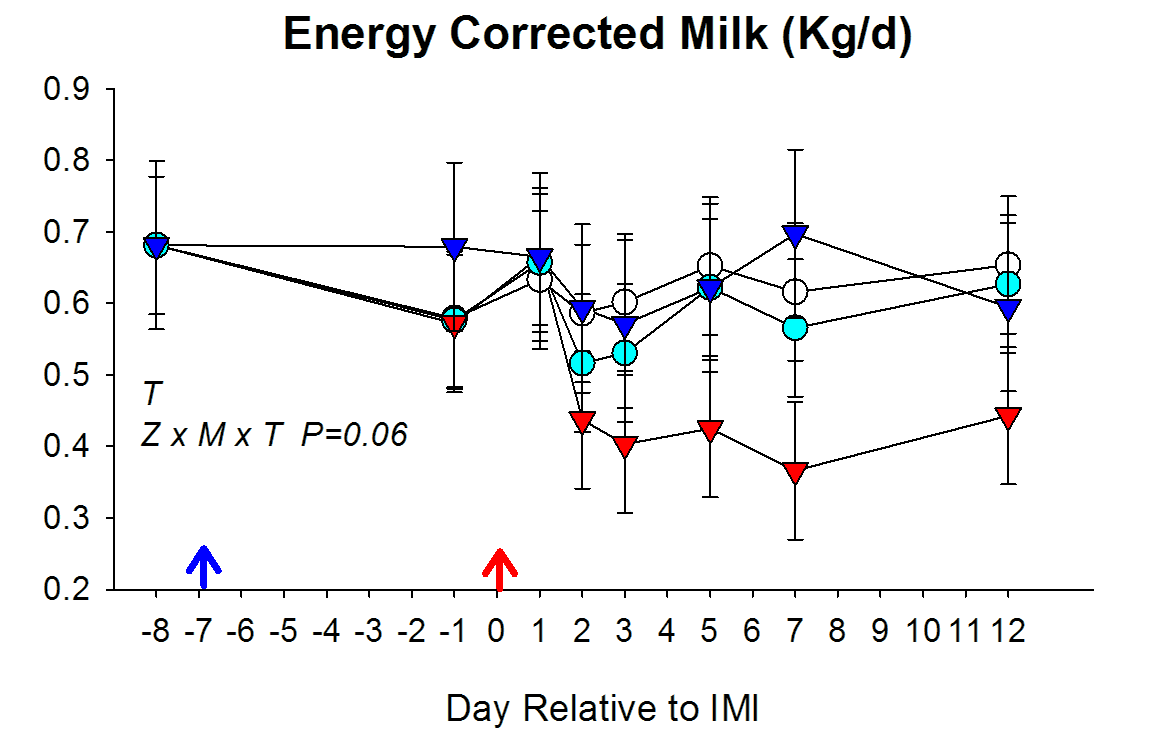


**Figure S6.** Energy corrected milk in goats receiving intramammary infusion (IMI) of *Strept. uberis* (M) or saline plus daily intrajugular injection of 2,4-thiazolidinedione (TZD) or saline (CTR). Significant (P≤0.05) effects and interactions are indicated in the graph (mastitis = M, Time = T, TZD = Z).

.


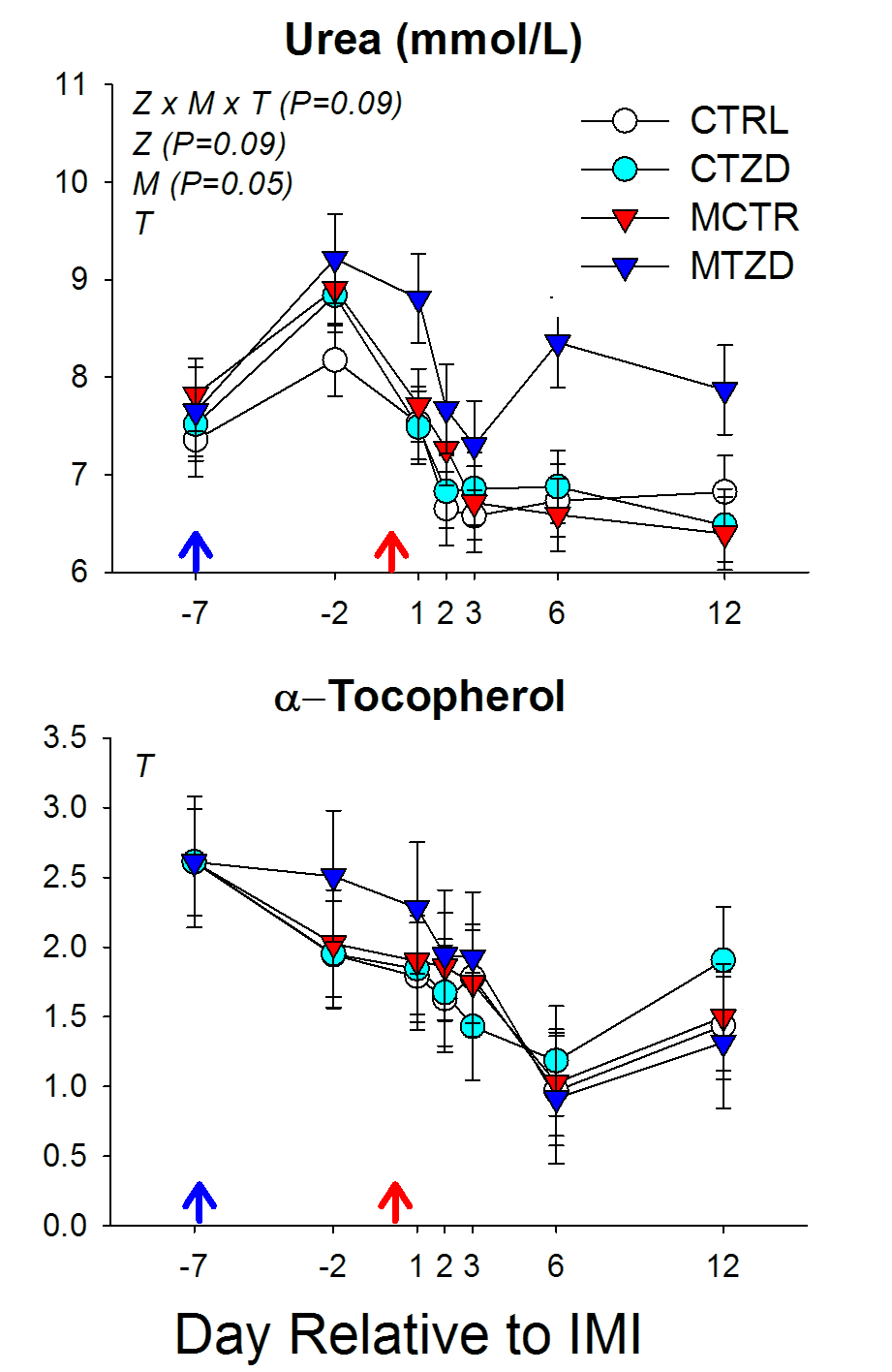


**Figure S7**. Plasma concentration of urea and α-tocopherol in goats receiving intramammary infusion (IMI) of *Strept. uberis* (M) or saline plus daily intrajugular injection of 2,4-thiazolidinedione (TZD) or saline (CTR). Significant (P≤0.05) effects and interactions are indicated in the graph (mastitis = M, Time = T, TZD = Z).

**
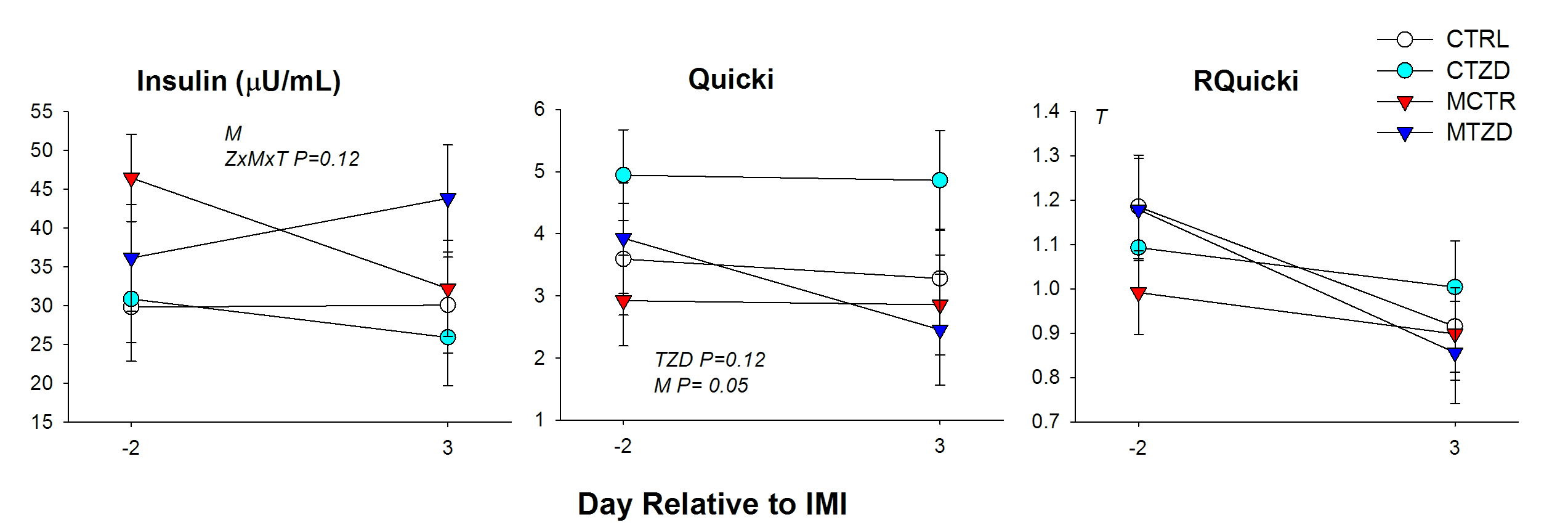
**

**Figure S8.** . Plasma concentration of insulin plus Quantitative Insulin Sensitivity Check Index (QUICKI) and the Revised QUICKI (RQUICKI) in goats receiving intramammary infusion (IMI) of *Strept. uberis* (M) or saline plus daily intrajugular injection of 2,4-thiazolidinedione (TZD) or saline (CTR). Significant (P≤0.05) effects and interactions are indicated in the graph (mastitis = M, Time = T, TZD = Z).


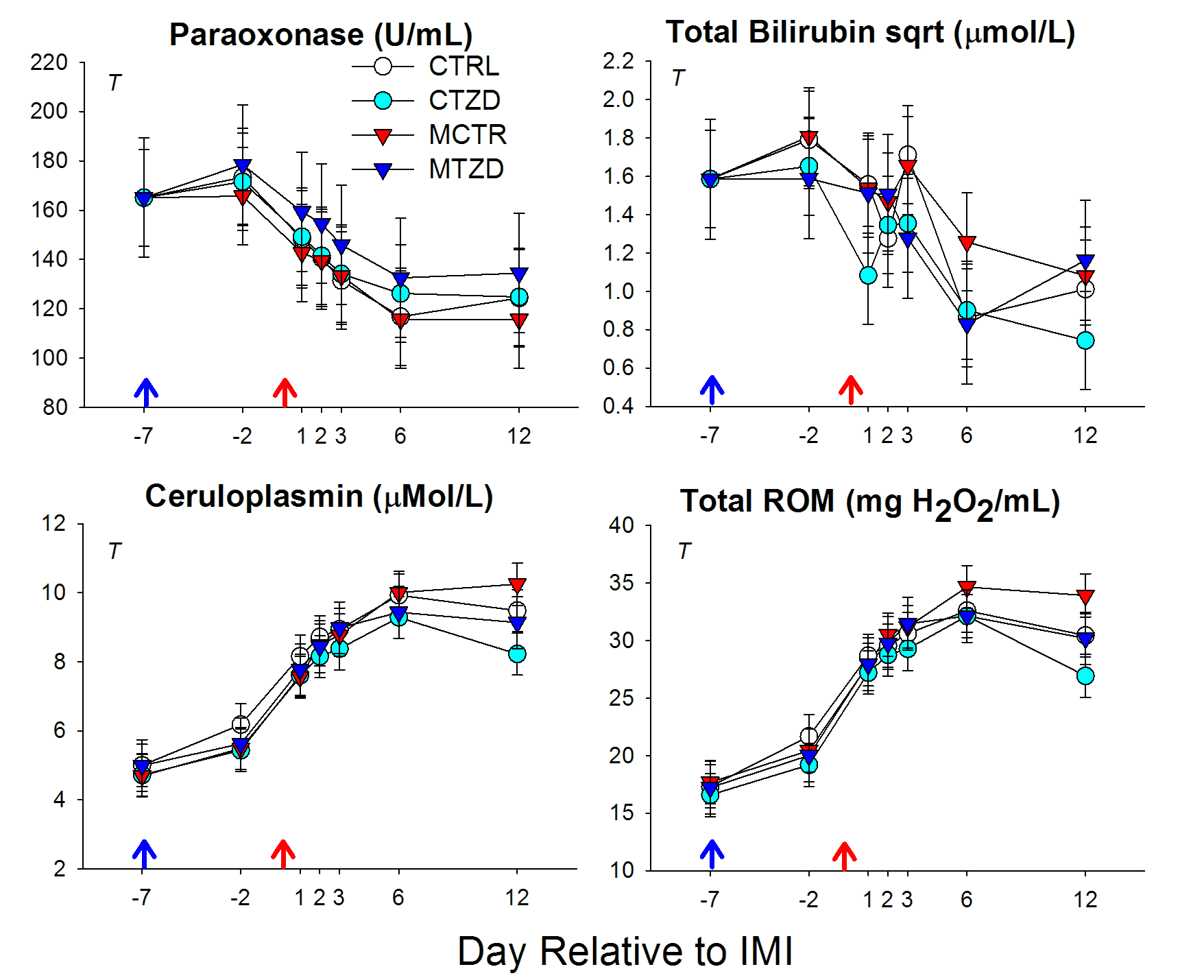


**Figure S9**. Plasma concentration of positive (ceruloplasmin) and negative (paraoxonase) acute phase reaction markers, total bilirubin as index of liver clearance, and total reactive oxygen metabolites (ROM) as index of oxidative stress in goats receiving intramammary infusion (IMI) of *Strept. uberis* (M) or saline plus daily intrajugular injection of 2,4-thiazolidinedione (TZD) or saline (CTR).

**
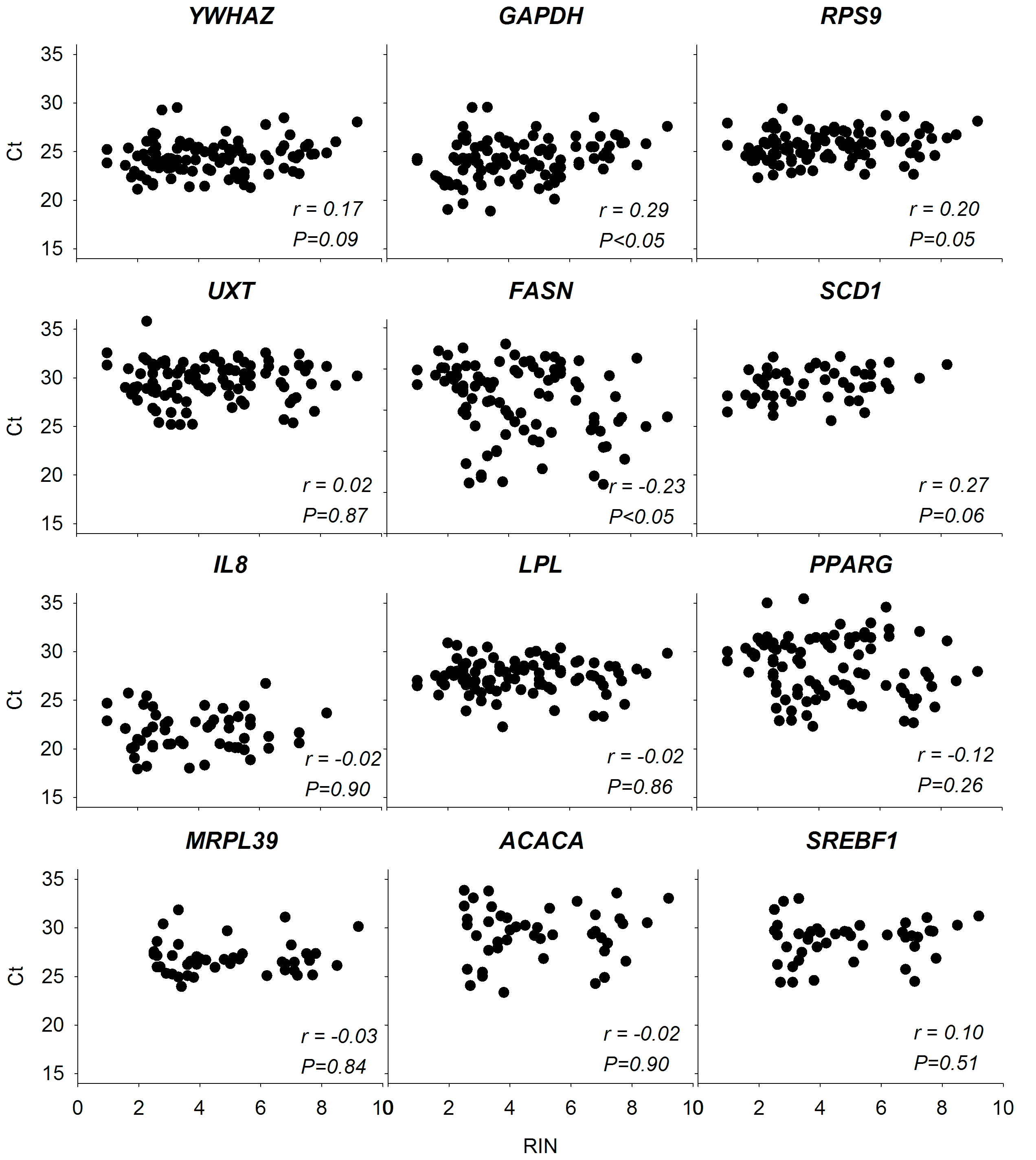
**

**Figure S10.** Pearson correlation between RNA Integrity Number (RIN) as calculated by Bioanalyzer and Ct values obtained by RTqPCR for all measured genes combining results from adipose and mammary epithelial cells. Reported in each graph are the value of the Pearson correlation coefficient r and its statistical significance.

**
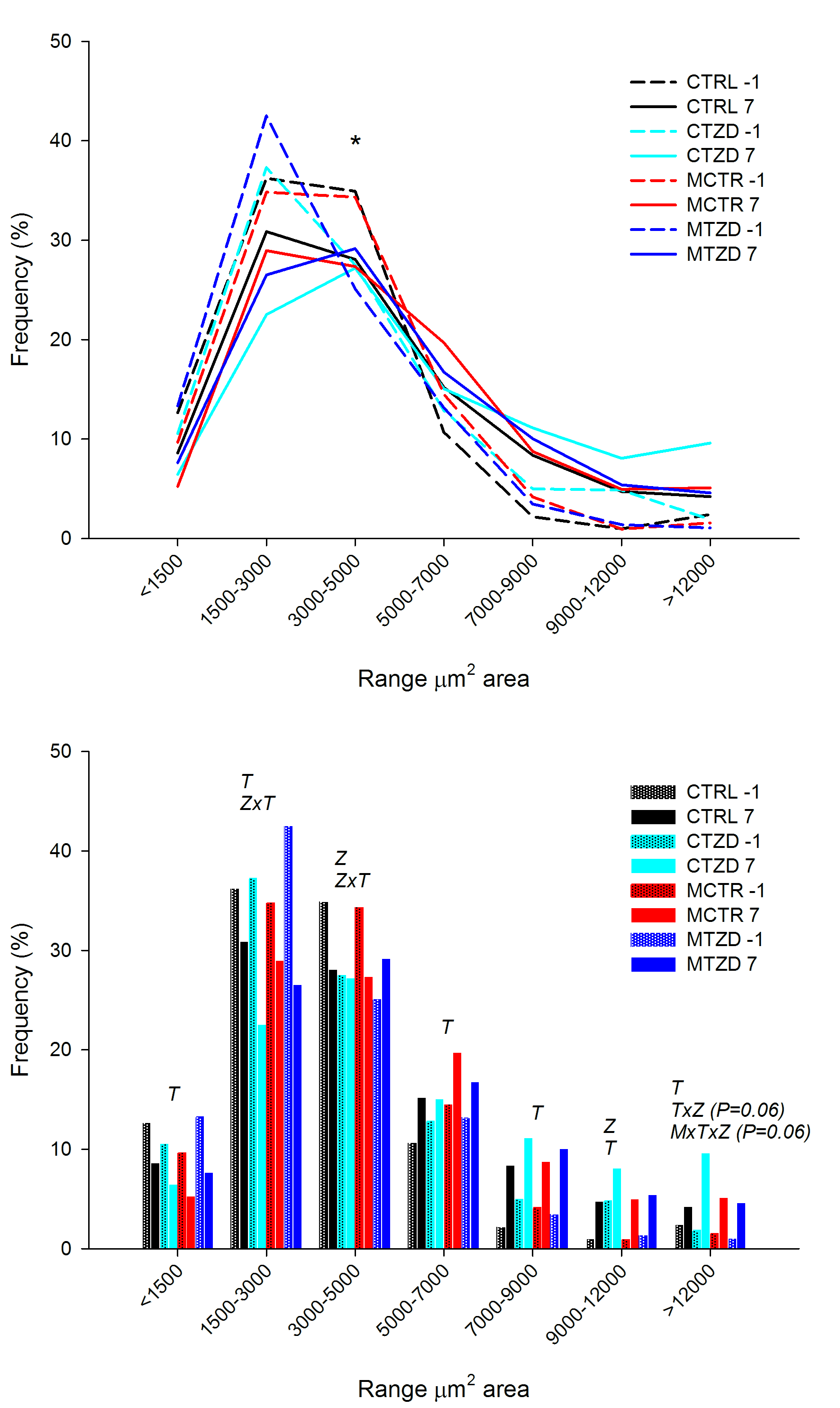
**

**Figure S11**. Adipocytes area in sub-cutaneous adipose tissue in goats receiving intramammary infusion (IMI) of *Strept. uberis* (M) or saline plus daily intrajugular injection of 2,4-thiazolidinedione (TZD) or saline (CTR) at -1 and 7 day post-IMI. Upper panel is the result of the statistical analysis considering all interactions, including adipose area range. The lower panel represents the analysis of each individual adipose area range for all groups of goats with both time points. Significant (P≤0.05) effects and interactions are indicated in the graph (mastitis = M, Time = T, TZD = Z).


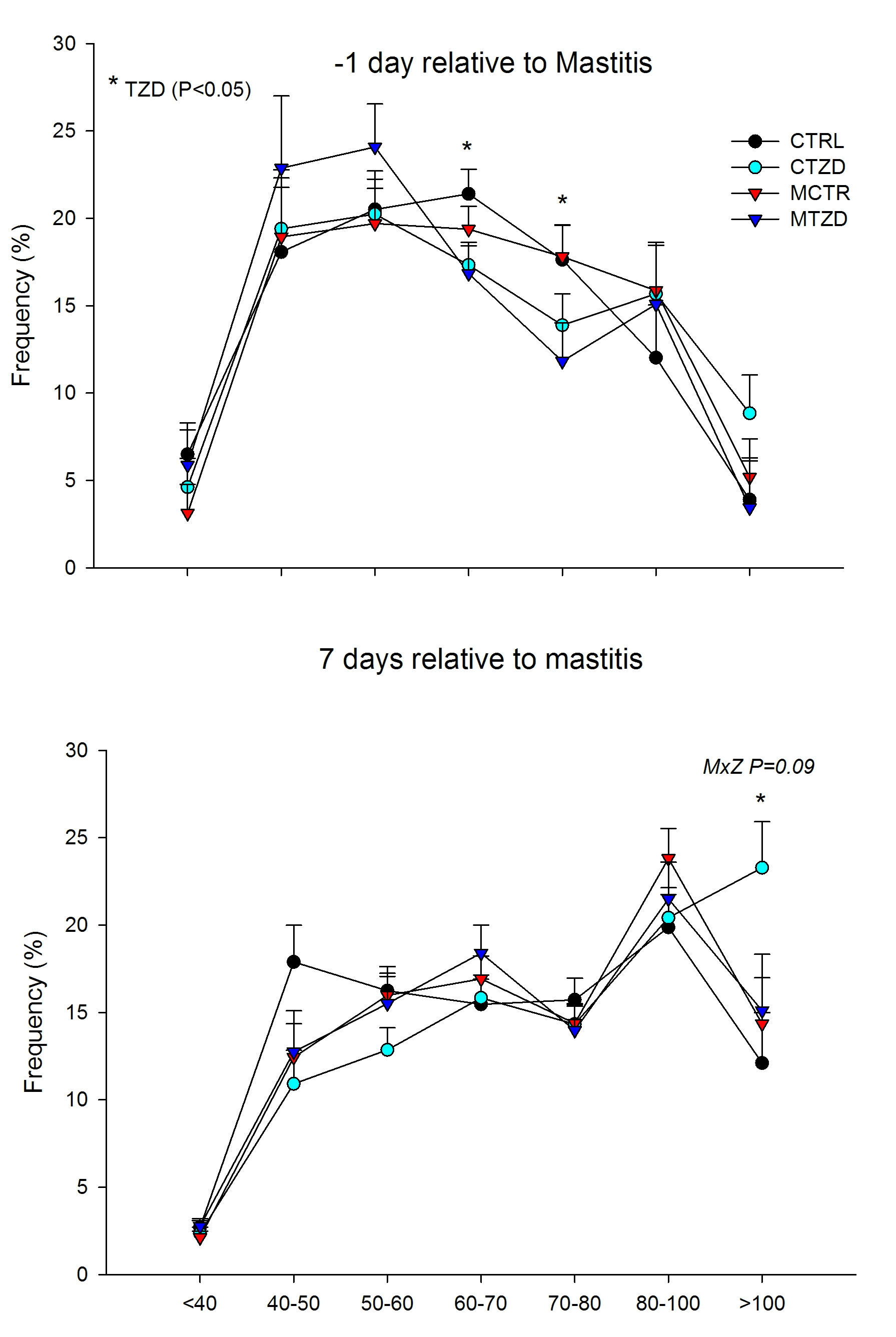


**Figure S12**. Diameter of adipocytes in sub-cutaneous adipose tissue in goats receiving intramammary infusion (IMI) of *Strept. uberis* (M) or saline plus daily intrajugular injection of 2,4-thiazolidinedione (TZD) or saline (CTR) at -1 (upper panel) and 7 (lower panel) day post-IMI. Significant (P≤0.05) effects are indicated in the graph (M = mastitis; Z = TZD) by*.


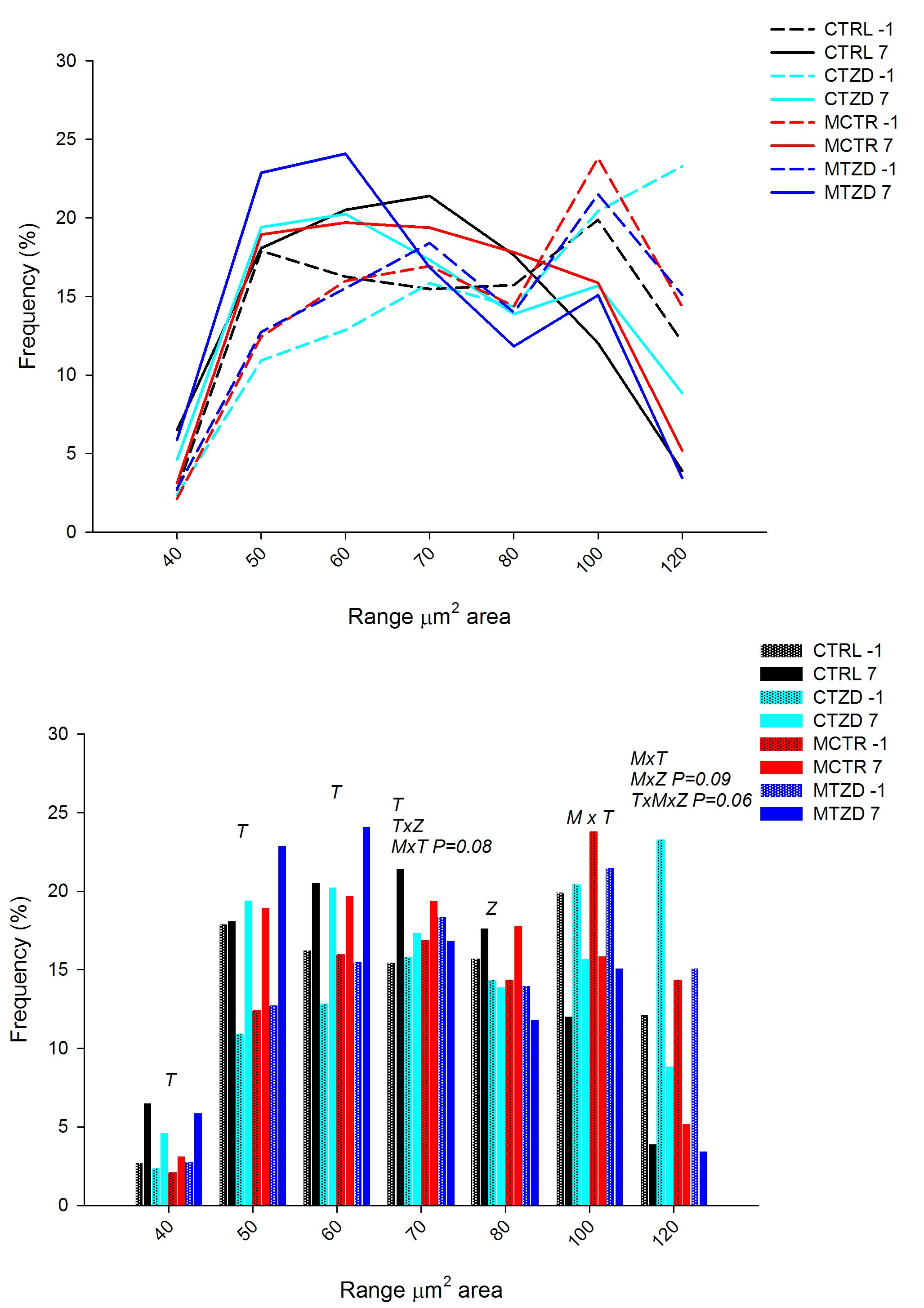


**FigureS13**. Adipocytes diameter in sub-cutaneous adipose tissue in goats receiving intramammary infusion (IMI) of *Strept. uberis* (M) or saline plus daily intrajugular injection of 2,4-thiazolidinedione (TZD) or saline (CTR) at -1 and 7 day post-IMI. Upper panel is the result of the statistical analysis considering all interactions, including adipose area range. The lower panel represents the analysis of each individual adipose area range for all groups of goats with both time points. Significant (P≤0.05) effects and interactions are indicated in the graph (mastitis = M, Time = T, TZD = Z).
